# Consistency within change: Evaluating the psychometric properties of a widely used predictive-inference task

**DOI:** 10.3758/s13428-024-02427-y

**Published:** 2024-06-06

**Authors:** Alisa M. Loosen, Tricia X. F. Seow, Tobias U. Hauser

**Affiliations:** 1 Max Planck UCL Centre for Computational Psychiatry and Ageing Research, London, UK; 2 Wellcome Centre for Human Neuroimaging, University College London, University College London, London, UK; 3 Center for Computational Psychiatry, Icahn School of Medicine at Mount Sinai, New York, NY USA; 4 Department of Psychiatry and Psychotherapy, Medical School and University Hospital, Eberhard Karls University of Tübingen, Tübingen, Germany; 5 German Center for Mental Health (DZPG), Tübingen, Germany

**Keywords:** Decision-making, Learning, Predictive inference, Test–retest reliability, Internal consistency, Psychometric qualities

## Abstract

Rapid adaptation to sudden changes in the environment is a hallmark of flexible human behaviour. Many computational, neuroimaging, and even clinical investigations studying this cognitive process have relied on a behavioural paradigm known as the predictive-inference task. However, the psychometric quality of this task has never been examined, leaving unanswered whether it is indeed suited to capture behavioural variation on a within- and between-subject level. Using a large-scale test–retest design (T1: *N* = 330; T2: *N* = 219), we assessed the internal (internal consistency) and temporal (test–retest reliability) stability of the task’s most used measures. We show that the main measures capturing flexible belief and behavioural adaptation yield good internal consistency and overall satisfying test–retest reliability. However, some more complex markers of flexible behaviour show lower psychometric quality. Our findings have implications for the large corpus of previous studies using this task and provide clear guidance as to which measures should and should not be used in future studies.

**Supplementary Information:**

The online version contains supplementary material available at 10.3758/s13428-024-02427-y.

## Introduction

Our ability to navigate the world depends on how successfully we respond to changes in our environment. In stable environments, we should rely on past experiences to guide our actions and beliefs and ignore (noisy) current deviations. However, flexibility is essential when we are in dynamic environments and exposed to sudden changes. Dynamic responses to our environment are a hallmark of adaptive behaviour, and these have been linked to specific neurocognitive learning mechanisms, including the adaptation of neural representations (e.g., Behrens et al., 2007; McGuire et al., 2014; Nassar, McGuire et al., 2019b; O’Reilly et al., 2013; Pearson et al., 2011) and changes in the brain’s functional connectivity (e.g., Kao, Khambhati et al., 2020a).

The predictive-inference task (often also referred to as the ‘helicopter task’) has been widely used to study such flexible behaviour. Pioneered by Nassar and colleagues (Nassar et al., 2010), it has been adapted in a number of variations (e.g., Bruckner et al., 2020; Jepma et al., 2016; Kao, Khambhati et al., 2020a; Krishnamurthy et al., 2017; McGuire et al., 2014; Nassar et al., 2012, 2016; Nassar, McGuire, et al., 2019b; Nassar et al., 2021; Nassar & Frank, 2016; Nassar & Troiani, 2020; Razmi & Nassar, 2020; Seow & Gillan, 2020; Vaghi et al., 2017). In this paradigm, participants are asked to predict the next position of a target that lands in a similar location for several trials. However, on some trials, the landing location suddenly shifts to a completely new position. To perform well in the task, participants must adapt flexibly to these sudden changes by altering their behaviour based on the new information while ignoring the information they received before the change. Tracking participants’ actions on each trial allows the characterisation of learning (Bruckner et al., 2020; Jepma et al., 2016; Nassar et al., 2010, 2016; Razmi & Nassar, 2020; Ritz et al., 2018) and the relevant arousal (Krishnamurthy et al., 2017; Nassar et al., 2012) and neural mechanisms (Kao, Khambhati et al., 2020a, Kao, Lee et al., 2020b, 2020c; McGuire et al., 2014; Nassar, Bruckner et al., 2019a; Nassar, McGuire et al., 2019b) associated with it.

Task behaviour has been captured both using behavioural indicators as well as Bayesian learner models (e.g., Nassar et al., 2010, 2016). The latter approach not only captures a quasi-optimal completion of a given task run but also formalises salient features of it, such as uncertainty (or relative uncertainty) and surprise (captured as change-point probability). These derived factors can then be linked to both human behaviour and neural changes during task completion. Differences in how task characteristics influence behaviour could provide important information on developmental changes and alterations in mental health disorders.

Cognitive flexibility is particularly relevant to psychiatric research, as it has been associated with several psychiatric disorders (Britton et al., 2010; Ceaser et al., 2008; Chamberlain et al., 2006, 2007; Geurts et al., 2009; Gu et al., 2008; Hauser et al., 2017; Loosen & Hauser, 2020; Morice, 1990; Nassar et al., 2021; Skvortsova & Hauser, 2022; Vaghi et al., 2017; Zhu et al., 2021). For example, a study using this predictive-inference task showed that patients with schizophrenia were prone to extreme forms of learning (i.e., little or complete behavioural adaptation to new evidence; Nassar et al. 2021), while patients with obsessive–compulsive disorder (OCD) have been shown to primarily over-emphasise new information at the cost of rashly discarding previously encountered evidence (Vaghi et al., 2017). These results suggest that different mechanisms may underlie cognitive inflexibility in different patient populations. However, to be able to draw such inferences about individual differences, the paradigm used to investigate them has to be psychometrically sound. Despite the great popularity of the predictive-inference paradigm, to our knowledge, the psychometric properties of the task measures have not yet been systematically investigated.

Low replicability has been identified as a challenge for the field (Open Science Collaboration, 2015) and low psychometric qualities are a major contributor to it (LeBel & Paunonen, 2011; Matheson, 2019). To test the foundation of the large body of research using this predictive-inference task, we believe it is thus critical to assess the psychometric quality of its measures. Recent studies in the field have recognised this need and have probed the psychometric qualities of several task measures, as well as analytical approaches to gain insight into their general and clinical use and the questions they may or may not be able to address (e.g., Brown et al., 2020; Mkrtchian et al., 2021; Pike et al., 2022; Pratt et al., 2021; Schaaf et al., 2023; Shahar et al., 2019; Waltmann et al., 2022). By doing so, researchers can gain a better understanding of the limitations and possibilities of the used tasks in addressing specific research questions.

The most prominent psychometric properties that a task must satisfy are internal consistency and test–retest reliability. Internal consistency quantifies the consistency of task measures across trials within a single task execution (Green et al., 2016; Matheson, 2019). High internal consistency stands for minimal confounding measurement noise. In contrast, test–retest reliability characterises the stability of a task measure over time. That is, the stability of the measure between one task administration and a second administration that follows after a predetermined time interval (Calamia et al., 2013; Matheson, 2019). This is essential when making inferences about stable neurocognitive traits and comparing variability between participants, as often done in psychiatric or pharmacological studies (e.g., Hauser et al., 2019; Michely et al., 2021; Mkrtchian et al., 2021). It is therefore crucial to assess both psychometric properties when drawing inferences about intra- and inter-individual differences (Green et al., 2016; Hedge et al., 2018; Matheson, 2019; Parsons et al., 2019).

In this study, we thus investigated the psychometric properties of this widely used predictive-inference task by conducting a large-scale, test–retest online study in the general UK public. Participants played the task twice with several months in between, which allowed us to quantify internal consistency and test–retest reliability. We show that while several ‘raw’ measures, such as confidence and learning rate before and after environmental changes, are mostly stable and reliable, others such as learning rate at the point of environmental change or the measures’ associations with Bayesian model predictions, show lower psychometric quality and should be used with caution.

## Methods

### Procedure

We conducted a large-scale online study in which participants played the predictive-inference task (Nassar et al., 2010) on two occasions. Data collection for the first time point (T1) took place at the end of April/ beginning of May 2020. At T1, participants reported their demographics and completed a cognitive ability assessment whose total score served as a proxy for IQ (Condon & Revelle, 2014) before playing the task. We re-contacted the participants to play the task again between mid-July and mid-August 2020 (time point 2; T2). They also reported their obsessive–compulsive (OC), anxiety and, depressive symptoms (cf. Questionnaires below) at T2. The mean time difference between T1 and T2 per participant was 81 days (min = 69 days, max = 104 days).

### Sample size estimation

As there was no prior data on the estimated reliability of the predictive-inference task, we powered our sample to be sensitive to the (relatively modestly sized) associations between psychiatric measures and task indicators. We based our power analysis (conducted in G*Power ; Faul et al., 2009) on the correlation between mean task confidence and OC symptoms in the sample reported by Seow and Gillan (2020) on trials that had the same hazard rate (the rate at which change-points (CPs) occurred) as we used here (H = 0.125; *r* = 0.272, *p* < 0.001). This analysis suggested our study required *N* = 190 participants to achieve 90% power at a *p* = 0.01 significance level.

### Participants

We recruited participants who were over 18 and living in the UK via the Prolific recruiting service (https://www.prolific.co/). All participants received a payment of £8.25/hour plus a bonus of up to £1 dependent on their task performance.

A total of 401 participants completed the study at T1. We excluded 19 participants because they failed at least one of two attention checks (instructed questionnaire answers). We additionally applied task-based exclusion criteria in line with previous studies (Seow & Gillan, 2020; Vaghi et al., 2017) to ensure good task data quality (cf. below in the task description and in the Supplemental Information). Based on these criteria, 52 additional participants were excluded. This resulted in a final T1 sample of 330 participants (187 females and 143 males; age: 34 ± 12.2 years). The T1 sample was larger than the sample size suggested by the power estimation as we expected attrition across both time points.

Participants from T1 were re-invited to take part in T2, from whom 260 completed the study a second time resulting in an overall retention rate of 79%. We again excluded participants because of failed attention checks (*N* = 14) and task exclusion criteria (*N* = 27). This resulted in a final T2 sample of 219 participants (119 females and 100 males; 35 ± 12.3 years).

### Material

We implemented all questionnaires using React JS libraries (https://reactjs.org/). The predictive-inference task was implemented in JavaScript.

#### The predictive-inference task

**Fig. 1:**
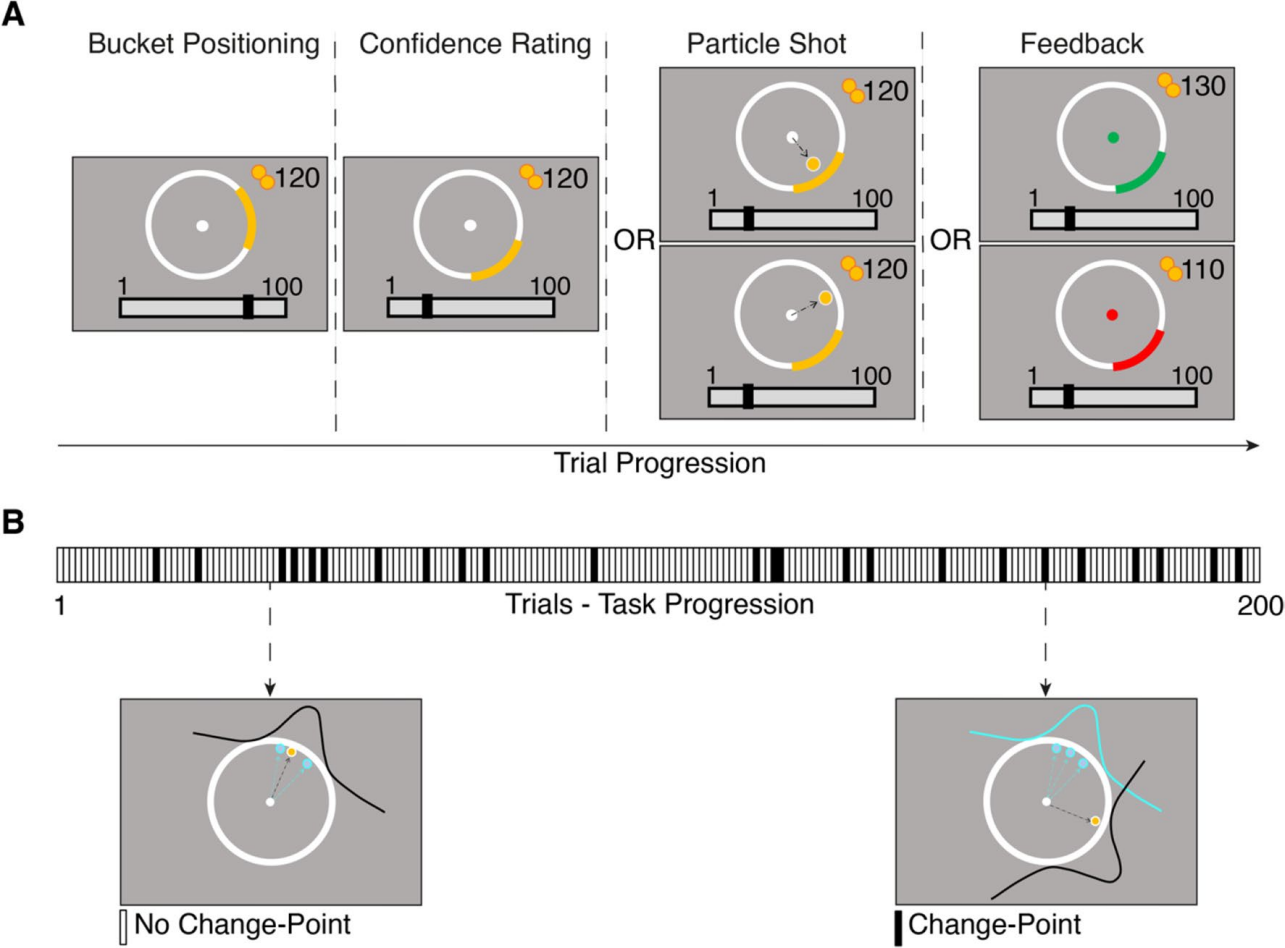
The Predictive-Inference Task. On each trial, participants placed a bucket (*yellow arc*) on the edge of a circle to catch a flying particle (**A**; first column from the left). Participants then indicated their confidence in catching the particle (**A**; second column from the left). The particles were launched from the centre of the circle to the edge (**A**; third column from the left). If the particle landed in the bucket, participants received ten points, and if the particle missed the bucket, they lost ten points (**A**; fourth column from the left). Participants completed a total of 200 trials, with approximately 24 change-points (CPs; **B**). The landing location of the particle was determined by sampling from a Gaussian distribution on each trial. The *dashed arrow lines* represent the particle's trajectory in the current (*black*) and past (*blue*) trials (**B**; lower left-hand side box). When a CP occurred, the mean of the Gaussian distribution abruptly shifted to another location on the circle (**B**; lower right-hand side box)

Participants played the predictive-inference task pioneered by Nassar and colleagues (Bruckner et al., 2020; Jepma et al., 2016; Kao, Khambhati et al., 2020a; McGuire et al., 2014; Nassar et al., 2010, 2012, 2016, Nassar, Bruckner et al., 2019a, 2021; Nassar & Troiani, 2020). We used the circular online version by Seow and Gillan (2020), an adaptation of the in-lab version utilised by Vaghi et al. (2017; cf. Fig. 1). We instructed participants to catch a particle flying from a circle centre to its edge by moving and placing a ‘bucket’ on this edge. The bucket's initial location was randomly determined on the first trial of the task. For all subsequent trials, the bucket was initiated at the position where the participant had placed it on the preceding trial. Participants used the left and right arrow keys to adapt the bucket’s position and the spacebar to confirm it. After placing the bucket, a confidence scale (ranging from 1 to 100) appeared below the circle. The confidence indicator was initially randomly placed at either 25 or 75, and participants adjusted it to report how confident they were that the particle would land in the positioned bucket. Subsequently, a particle flew from the circle centre to the edge (feedback). If the particle landed within the bucket, the bucket turned green for 500 ms, and the participant gained ten points. If the particle landed outside the bucket, it turned red, and the participant lost ten points. Accumulated points were presented in the upper-right corner. These points were converted into a bonus payment at the end of the task (maximum £1). Confidence ratings were not incentivised, however, to ensure active usage of the scale, we followed a procedure consistent with prior research on this task (e.g., Seow & Gillan, 2020). Specifically, if participants left their confidence ratings at the default score (i.e., the position to which the confidence indicator was randomly set at the start of the trial) for more than 70% of the first 50 trials, the task was reset. This was the case for 6 participants at T1 and 2 participants as T2. In addition, we excluded participants who had left the confidence indicator at the initial anchor point on more than 60% of the trials (T1: *N* = 14; T2: *N* = 4), if the initial confidence anchor correlated more than 0.5 with their logged confidence rating (T1: *N* = 17; T2: *N* = 17) and if their mean confidence ratings on trials following correct trials were significantly lower than their mean confidence ratings on trials following incorrect trials (T1: *N* = 19; T2: *N* = 6; cf. Supplemental Information).

On each trial, the particle’s landing location on the circle edge was sampled independently from a Gaussian distribution with a standard deviation of 12 degrees. The mean of this distribution remained the same until a CP trial took place. At the CP, the mean of the generative distribution determining the landing position shifted to a new position. This new position of the mean was re-sampled from a uniform distribution U(1,360) (i.e., number of points on the circle; cf. Fig. 1C). The probability of a CP occurring on each trial was determined by the hazard rate of 0.125 and was stable throughout the entire task. Participants had to observe into which part of the circle edge the particle fell after a CP had happened and adapt their behaviour accordingly. They completed 200 trials in total (which results in approximately 24 CPs), divided into four blocks of 50 trials separated by self-timed short breaks. To ensure that participants understood the task, they had to complete ten practice trials after reading the instructions. They then had to answer five questions on the task instructions correctly before starting the actual game and were asked to start again from the instructions if they failed one or more of these questions.

#### Questionnaires

We assessed obsessive-compulsive (OC) symptoms using the Obsessive–Compulsive Inventory-Revised (OCI-R; Foa et al., 2002), as used by previous studies using the present task (Seow & Gillan, 2020). To control for potential confounds in the OCI-R score caused by the COVID-19 pandemic that was taking place during data collection (Banerjee, 2020; Loosen et al., 2021; Tanir et al., 2020), we computed an additional score controlling for items of high relevance to COVID-19 (cf. Supplemental Information). We additionally measured anxiety and depression symptom scores using the Hospital Anxiety and Depression Scale (HADS; Zigmond & Snaith, 1983), which consists of two subscales (anxiety, depression) that are evaluated separately.

### Quantitative analysis

We pre-processed and analysed data in MATLAB 2021b (MathWorks) and R, version 3.6.2 via RStudio version 1.2.5033 (http://cran.us.r-project.org)

#### Computational models

##### Learning rate characterising human behaviour

Following most recent approaches in the literature (e.g., Nassar, Bruckner et al., 2019a), we characterised participants’ behaviour by computing their prediction error (PE^*h*^), the circular distance between the bucket centre (*b*) and the location where the particle landed (*X*) on trial *t*

1 $${PE}_{t}^{h} = {X}_{t}-{b}_{t}$$

In addition, we also derived a human learning rate (e.g., Seow & Gillan, 2020; Vaghi et al., 2017) defined as the action-update (i.e., change in bucket position from trial *t* to *t*+1 computed as a circular distance) divided by the PE^*h*^ on trial *t* (LR^*h*^):

2 $${LR}_{t}^{h} = \frac{{b}_{t+1}-{b}_{t}}{{PE}_{t}^{h}}$$

An LR^*h*^ of 1 would mean that newly encountered information (most recent particle falling position) overshadows preceding information.

##### Normative factors characterising task fluctuations

We also characterised the task dynamics using a reduced quasi-optimal Bayesian learner as used in previous studies (Nassar et al., 2016). This model is applied to task data and PE^*h*^s experienced by participants while adjusting its predictions Bayes-optimally. Normative factors derived from the learner capture task characteristics thought to influence participants’ behaviour and confidence ratings in a changing (task) environment: For each trial, the resulting factors captured (1) the approximation of the probability that a change-point had occurred (change-point probability; CPP; alternatively referred to as ‘surprise’ in the literature) and (2) relative uncertainty in the belief about the mean of the generative distribution determining the particle landing location (relative uncertainty; RU). RU is comprised of noise and uncertainty in the estimation itself (due to unreliability of the current estimate of the mean). While noise refers to the inherent randomness in predicting a single sample from a known generative distribution, the second source of uncertainty (i.e,. unreliability of the current estimate of the mean) decreases with more data collected (akin to gain in a Kalman filter). The reduced quasi-optimal Bayesian learner and all normative factors are further described in detail in the Supplemental Information and Supplemental Figs. 6 and 7 link raw behavioural measurements to Bayesian factors.

To evaluate the consistency and reliability of the relationship between this Bayesian learner and participants' behaviour, we utilised regression models (cf. below) and analysed the psychometric properties of the resulting beta values, which have been frequently used in relevant studies (e.g., McGuire et al., 2014; Nassar et al., 2016, Nassar, Bruckner et al 2019a, Nassar, McGuire et al. 2019b; Seow & Gillan, 2020; Vaghi et al., 2017).

Following previous studies using this task (Seow & Gillan, 2020; Vaghi et al., 2017), trials where the LR^*h*^ exceeded the 99th percentile of all participants LR^*h*^ or where PE^*h*^ = 0 were assumed to be unrelated to error-driven learning (Nassar et al., 2016) and were thus excluded from the analyses that were based on normative factors derived from the Bayesian learner.

#### Psychometric properties

As the processing of environmental changes lies at the core of this task, reliability of measurements relative to these changes (i.e., CPs) is of most importance. We, therefore, computed all psychometric scores relative to CPs to ascertain whether measures robustly captured responses to environmental changes. To capture the stability of the raw behavioural measures (i.e., LR^*h*^ and confidence) relative to the CPs, we computed all psychometric estimates separately for each trial preceding CPs (4 to 1 trials before), the CP trial itself, and trials following CPs (1 to 4 trials after). To investigate the psychometric properties of the measures during stable and unstable phases of the task, we analysed the properties of the average scores across the two trials immediately before versus after CPs respectively. We also investigated how many CPs participants had to experience until the main variables reached a satisfactory level on both psychometric measures and report the test–retest reliability across all trials for completeness (cf. Supplemental Information).

Following this approach, we examined the psychometric properties of variables that are widely used. Specifically, we assessed the consistency and reliability of the median LR^*h*^ and confidence. We chose the median due to their skewness and extreme values in the LR^*h*^, resulting from variations in particle landing location and the corresponding differences in PE magnitude.

##### Internal consistency

We used the split-half approach (Green et al., 2016) to estimate the internal consistency of the task. For each time point, we split the task data into two sub-datasets (odd and even; cf. below) and computed all measurements for them separately. We then measured the internal consistency by computing the Pearson correlation corrected with the Spearman–Brown formula (Brown, 1910; Spearman, 1910) between measures gained from the odd and even datasets. The Spearman–Brown correction was computed using the *psych* package in R (Revelle, 2020). It extrapolates the reliability estimation from the length of the sub-dataset to the length of the entire dataset (de Vet et al., 2017). In line with conventions in the field (Shahar et al., 2019), we categorised internal consistency coefficients below 0.5 as ‘low’, coefficients between 0.5, and 0.7 as ‘moderate’ and coefficients above 0.7 as ‘good’.

When investigating the internal consistency of the raw measures (e.g., LR^*h*^, confidence) relative to the CPs (before, at and after), we created the odd and even sub-datasets by splitting the full dataset based on CPs and their associated trials (i.e., all trials following a CP before the next CP occurred) since an equal number of CPs in one versus the other sub-dataset was essential. We then estimated the internal consistency of the raw measurements of interest (i.e., LR^*h*^ and confidence) on all trials ranging from four trials before to four trials after CPs individually. For the sake of completeness, we additionally computed internal consistency across all CP relative trials.

We also estimated the internal consistency of beta coefficients gained from regression models that linked behavioural measurements and normative factors derived from the Bayesian learner. We thereby followed the standard splitting procedure dividing odd and even trials into odd and even regression datasets. We then computed regression models based on these two datasets and approximated the consistency of the resulting beta values. This enabled us to approximate the extent to which these two regression models revealed the same associations.

##### Test–retest reliability

We estimated the task’s temporal (test–retest) reliability using ICC as implemented in the R *psych* package (Revelle, 2020). The ICC estimates the agreement between measurements while capturing differences in means of the compared scores (e.g., systematic shifts due to training effects). This distinguishes it from other measures such as Pearson correlation, making ICC more conservative in its results, although often yielding similar overall conclusions (Koo & Li, 2016). The ICC is the ratio of variability between participants to the total variability, including participant and error variability. Measures with low between-participant relative to between-time-point (within-participant) variability may be considered suitable for experimental designs, but unsuitable for correlational or individual-difference approaches (Hedge et al., 2018). As per recommendations by Shrout and Fleiss (1979), we chose the ICC(2,1) score, which represents the absolute agreement ICC score for single scores. This score takes into account a random effect of time, accounting for potential learning effects between testing sessions (Parsons et al., 2019) and a random effect of participant. We also explored the ICC(3,1) scores, which has a fixed effect of time and revealed similar scores, leading to the same overall conclusions. Following the conventional criterion (Koo & Li, 2016), we categorised ICC scores below 0.5 as ‘low’, between 0.5 and 0.75 as ‘moderate’, and above 0.75 as ‘good’.

We investigated the stability of the different regressions’ weights within participants across time by computing the ICC between the random slope coefficients at T1 and T2. To do so, we re-run the T1-models only including participants that had also completed T2.

#### Regression and correlation models

To characterise the link between task characteristics and behavioural characteristics (i.e., action-update and confidence), we ran several regressions that were fit separately for each participant, and tested the mean coefficients against zero at the group level using a two-sided *t* test. The action-update model aimed to investigate the modulation of error-driven learning by task variables, and thus all predictors (except for PE^*h*^ itself) were implemented as an interaction effect with PE^*h*^ and mean-centred following previous research (McGuire et al., 2014; Nassar, Bruckner et al., 2019a; Nassar et al., 2010, 2016; Seow & Gillan, 2020; Vaghi et al., 2017). To account for the circular character of the dependent variable, the model relied on a circular (i.e., Von Mises) error distribution around the predicted action-updates. To estimate the circular regression, we followed Nassar and colleagues’ (Nassar, Bruckner et al 2019a, Nassar, McGuire et al. 2019b) approach using the *fmincon* optimisation tool in MATLAB to derive maximum posterior coefficients for each subject, which were regularised by using a weak Gaussian zero-centred priors with a width of 5. We additionally ran linear control models used in previous studies (Seow & Gillan, 2020; Vaghi et al., 2017) that are reported in the Supplemental Information.

We also ran linear regression models predicting confidence on trial *t* based on regressors at trial *t-1*. These regressors were the *z*-scored absolute PE^*h*^ (i.e., shortest, total distance between the bucket centre and the particle landing location) and the normative factors CPP and RU as well as participants’ binary accuracy denoted as ‘Hit’.

In this task implementation we and others (Vaghi et al., 2017) used, changes in CPP were primarily driven by PE^*h*^ (cf. Supplemental Information; McGuire et al. 2014), and thus the two variables were highly correlated (T1*: r*(63879) = 0.931, *p* < 0.001; T2: *r*(42303) = 0.930, *p* < 0.001). Due to this co-linearity, we also ran control regression models with each predictor in separate models that replicated the below reported main findings (cf. Supplemental Information for similar and extended regression models).

#### Link to psychiatric questionnaire scores

Task reliability is of particular relevance when making inferences about inter-individual differences (Hedge et al., 2018) and its lack may result in spurious or/and inconsistent findings. Previous studies have used the present predictive-inference task to investigate behavioural differences between patients with OCD and control participants without a mental health diagnosis (Vaghi et al., 2017) as well as along psychiatric dimensions (Seow & Gillan, 2020). Seow and Gillan (2020) showed that decreased action-confidence coupling (i.e., coupling of confidence and action-update) was associated with multiple psychiatric questionnaire scores such as obsessive–compulsive (OC), anxiety (before Bonferroni correction), and depressive symptoms in the general public. On a transdiagnostic level, the compulsivity dimension showed a selective negative association with the confidence-action coupling. A similar decoupling had previously been found in patients (Vaghi et al., 2017). Despite this apparent similarity, the driving factors were dissimilar across the studies. While the transdiagnostic dimension of compulsivity was linked to an elevated confidence (Seow & Gillan, 2020), the patient study identified higher learning rates as the underpinning factor (Vaghi et al., 2017). As these findings seemed partially contradictory, we attempted to replicate them in our sample at the second time point.

## Results

To examine the psychometric properties of the predictive-inference task (cf. Fig. 1), 219 participants played the circle-version of the task (Seow & Gillan, 2020; Vaghi et al., 2017) at two time points (T1 and T2). We first assessed the reliability of the behaviour-derived learning rate (LR^*h*^) and the trial-by-trial confidence ratings and subsequently probed more complex associations with predictions of a quasi-optimal Bayesian learner.

### Learning in a changing environment

**Fig. 2:**
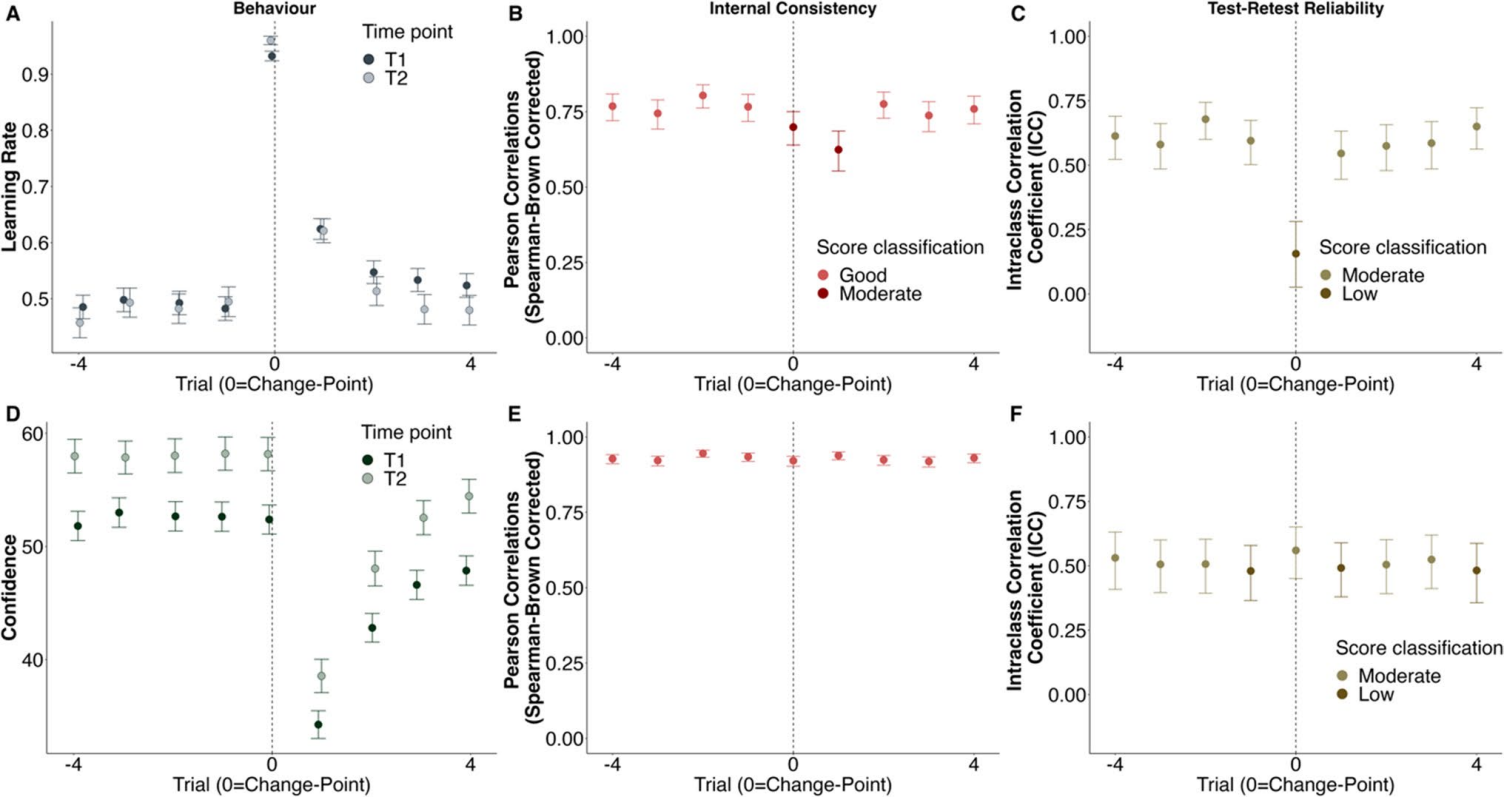
Behavioural and psychometric properties of human learning rate and confidence. Participants’ learning rates (LR^*h*^) at time point 1 (T1;* N*_*T1*_* =* 330) and 2 (T2;* N*_*T2*_ = 219) were highest at the change-point (CPs; *vertical dashed line*) and decreased afterwards back to their pre-CP levels (**A**). Accordingly, confidence ratings dropped after a CP and increased back to pre-CP levels afterwards (**D**). Internal consistency (Spearman–Brown corrected Pearson correlations) for the median LR^*h*^ was moderate to good at all investigated trials before, at, and after CPs (here displayed for T1; **B**). Test–retest reliability measured by ICC-scores between median LR^*h*^s at T1 and T2 were moderate before and after the CP but low at the CP itself (**C**). Median confidence showed good internal consistency (**E**) and low to moderate test–retest reliability across all trials (**F**). Behaviour and internal consistency for both measures are displayed for T1 only, but T2 statistics show similar results. *Error bars* represent standard errors in A & D and  95% confidence intervals in the remaining plots.

When looking at how trial-wise LR^*h*^ fluctuated relative to CPs, we found that CPs were associated with an increase in median LR^*h*^ (cf. Fig. 2A; cf. Supplemental Fig. 1 for a display of the overall LR^*h*^ distribution). This is in line with previous studies using different versions of the task (Jepma et al., 2016; McGuire et al., 2014; Nassar et al., 2010; Vaghi et al., 2017). The LR^*h*^ then decreased and levelled off after a few trials. This shows that when participants encountered an unexpected particle location due to a CP, they reacted to the novel location information by updating the bucket position more. This was underlined by a significant difference between the average LR^*h*^ at the CP itself and the average LR^*h*^ on the subsequent four trials, capturing the influence of CPs on the LR^*h*^ (T1: *t*(454.62) = 18.062,* p* < 0.001; T2: *t*(258.72) = 17.614,* p* < 0.001). Thus, participants seem to appropriately react to large changes in the environment as novel information has a high influence on their actions.

#### Psychometric properties

##### Internal consistency

We next tested the psychometric properties of the LR^*h*^, first estimating how consistent the median LR^*h*^ was at each time point and found that the internal consistency was moderate to high at the investigated trials at T1 and T2 (*r*_*SB*_ $\ge 0.624$; cf. Fig. 2B, Supplemental Table 1 and Supplemental Fig. 3A). This means that the LR^*h*^ and its fluctuation relative to environmental changes were consistent within participants. This supports the notion that this task can adequately capture how humans adapt to changing environments. We also found that mean LR^*h*^ (computed based on values bounded between 0 and 1), demonstrated similar internal consistency (data not reported here) to the median LR^*h*^ of the unbounded dataset. Moreover, the internal consistency of LR^*h*^ in stable (across the two trials before CPs) versus unstable phases (across the two trials after CPs; cf. Methods) was on a similar level (stable: T1: r_*SB*_ = 0.880, 95% CI [0.871, 0.887]; T2: *r*_*SB*_ = 0.912, 95% CI [0.905, 0.920]; unstable: T1: *r*_*SB*_ = 0.856, 95% CI [0.846, 0.865]; T2: *r*_*SB*_ = 0.874, 95% CI [0.863, 0.884]).

To capture the consistency of the CP-driven adaptation of the LR^*h*^ we also examined the internal consistency of the difference in average LR^*h*^ at the CPs itself versus at the trials after CPs. This difference score showed good internal consistency at both time points (T1: *r*_*SB*_ = 0.797, 95% CI [0.754, 0.833]; T2: *r*_*SB*_ = 0.856, 95% CI [0.816, 0.888]), demonstrating that participants consistently changed their LR^*h*^ in correspondence to changes in the task.

Because internal consistency is critically dependent on measurement noise, which in turn is affected by the number of trials in a task, we conducted an additional analysis investigating the number of change-points that were necessary to reach a satisfying consistency. To do so, we assessed the internal consistency under a reduced number of CPs (i.e., from six CPs until full task length with 24 CPs; cf. Supplemental Information). We found that on trials before the CP, the LR^*h*^ reached a moderate internal consistency after ~ 12 CPs, and a good consistency after ~ 24 CPs (i.e., r_*SB*_ $\ge$ 0.70). Similarly, median LR^*h*^ after the CP reached good internal consistency at ~ 21 CPs except for the LR^*h*^ on the first trial after the CPs, which stayed on a moderate level throughout and the CP-trial itself, which reached a good internal consistency at 24 CPs (cf. Supplemental Fig. 4A-B). This suggests that our task length totalling 24 CPs was appropriate for our hazard rate (H). Although consistency of LR^*h*^ never reached a good score at some trials the overall internal consistency is nonetheless convincing.

##### Test–retest reliability

Next, we estimated how much the LR^*h*^ scores from the first time point corresponded to the LR^*h*^ at the second time point ~3 months later, as indicated by their intra-class correlation (ICC). This analysis showed that the test–retest reliability at the trials before and after the CPs of the LR^*h*^ was moderate (ICC $\ge$ 0.545; cf. Fig. 2C; cf. Supplemental Table 1). Analyses based on the mean LR^*h*^ (bounded between 0 and 1) again showed similar psychometric properties (data not reported here). Thus, LR^*h*^ before and after CPs were relatively consistent from T1 to T2, suggesting suitability for studying stable individual differences. LR^*h*^ in stable versus unstable task phases was also moderately reliable (stable: ICC = 0.667, 95% CI [0.641 0.691]); unstable: ICC = 0.661, 95% CI [0.634, 0.687]). In line with the results above, test–retest reliability of mean LR^*h*^ was substantially lower (ICC $\le$ 0.286) supporting the investigation of median LR^*h*^s in the task.

The main exception was the LR^*h*^ at the CPs itself, which had a low test–retest reliability (ICC = 0.156, 95% CI [0.026, 0.284]). To examine this further, we looked at the total variance as decomposed for the calculation of the ICC-score and observed that the error variance was high (83%), while the between-participant variance was only 16% and between-time-point variance marginal (1%). Further inspection of the LR^*h*^ at the CP revealed that all LR^*h*^s were close to 1 for all participants, meaning that participants had very similar LR^*h*^s at the CPs (cf. Supplemental Fig. 3A). This is because all participants adapted their bucket position similarly strongly after a CP, which may be expected in this task. However, this also means that because the behaviour is very similar across participants, the CP-LR^*h*^ should not be used to assess individual differences.

The examination of the test–retest reliability of the CP-driven adaptation of the LR^*h*^ (i.e., difference between the average LR^*h*^ at the CPs itself versus at the trials after CPs), however, showed that the fluctuation was moderately reliable across time (ICC = 0.640, 95% CI [0.512, 0.729]). This indicates suitability for investigating differences across participants.

We again examined how this was linked to the number of trials and found that test–retest reliability of the trials before the CPs reached a moderate level (ICC = 0.50) after ~ 13 CPs (cf. Supplemental Fig. 4C-D). Overall, we found that the LR^*h*^ before and after CPs were sufficiently consistent across time, given the implemented number of CPs, but the LR^*h*^ at CP itself was too homogeneous and therefore not capable of differentiating between individuals. Moreover, the reported LR^*h*^ findings remained true when conceptualizing LR^*h*^ based on the unsigned, shortest linear version of action-update and PE^*h*^ as done by former studies (as in Seow and Gillan 2020; Vaghi et al. 2017) instead of the circular distance (cf. Supplemental Information).

### Dynamic decision confidence

To measure participants’ subjective uncertainty in their estimate (positioning of the bucket), we asked them to indicate at each trial how confident they were that their positioned bucket would catch the particle. Ideally, after a sudden change (CP), participants’ confidence should be low as they have little evidence as to where the particle will fall next (Nassar et al., 2010). Once the environment is more stable again, and particles on subsequent trials fall into nearby positions, confidence in their estimate should increase.

Guided by this prediction, we thus investigated the development of confidence ratings relative to CPs. We indeed saw that CPs were followed by a drop in confidence, which then rose again as trials passed (cf. Fig. 2D). This means, when a sudden change in the environment occurred and when participants had little information as to where the next particle would fall, they were uncertain as to whether their bucket would catch it. Subsequently, as they acquired more information regarding the new falling position with passing trials, they became more confident. This also occurred in previous studies using this or other adaptations of the predictive-inference task (e.g., Nassar et al. 2010; Vaghi et al. 2017).

#### Psychometric properties

##### Internal consistency

We analysed the internal consistency of confidence ratings and found that it was good across all trials, from four trials before to four trials after CPs at both time points (all *r*_*SB*_ $\ge$ 0.913; cf. Fig. 2E, Supplemental Table 1 and Supplemental Fig. 2B) and in stable (T1: r_*SB*_ = 0.964, 95% CI [0.961, 0.966]; T2: *r*_*SB*_ = 0.957, 95% CI [0.953, 0.960]) versus unstable (T1: r_*SB*_* =* 0.960, 95% CI [0.957, 0.963]; T2: *r*_*SB*_ = 0.958, 95% CI [0.954, 0.961]) task phases.

This level of internal consistency was, moreover, reached at all trials after only ~ 7 CPs (cf. Supplemental Fig. 4E-F), thereby only requiring approximately 56 trials with our hazard rate. This means, participants rated their confidence consistently throughout the game, on trials before, at, as well as after the CPs, and good internal stability was reached quickly.

##### Test–retest reliability

Investigating whether participants’ confidence ratings were consistent across time, we found mostly moderate test–retest reliabilities for all trials, before, at, and after CPs, (ICC $\ge$ 0.480; cf. Fig. 2F, Supplemental Fig. 3B and Supplemental Table 1). This level of test–retest reliability was reached after ~ 16 CPs (cf. Supplemental Fig. 4G-H). Test–retest reliability of average confidence ratings in stable versus unstable phases was also moderate (stable: ICC = 0.521, 95% CI [0.461, 0.574]); unstable: (ICC = 0.533, 95% CI [0.475, 0.584]).

While this means that confidence is reliable enough to be used to compare individual differences, it might be advisable to use more CPs than for LR^*h*^-investigations, which showed a better test–retest reliability (with the exception of the CP-trials) might be advisable.

### Lower confidence is positively linked to updates of the bucket position

**Fig. 3:**
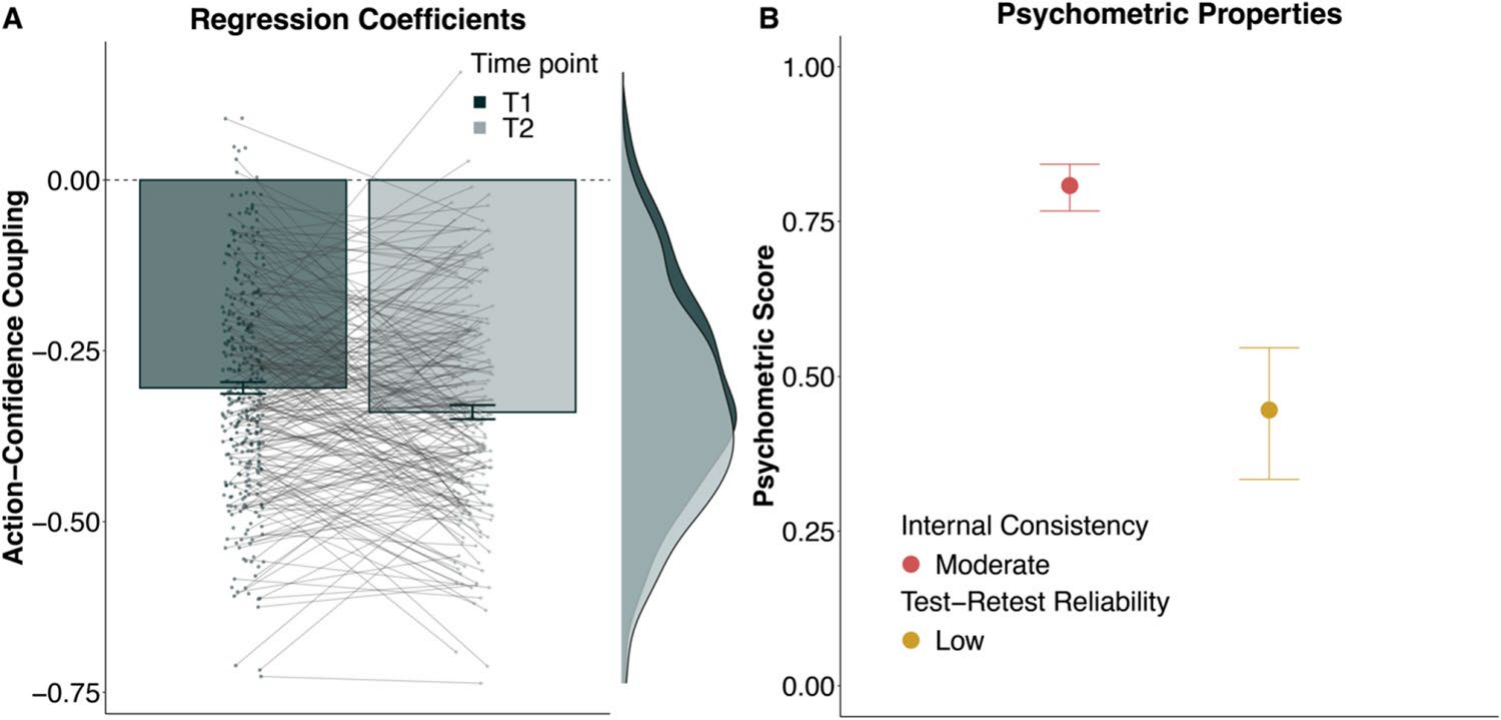
Relationship between action-update and confidence. We found a negative relationship between confidence and action-update (adaptation of the bucket position from one trial to the next) at both time points (N_T1_ = 330; N_T2_ = 219) showing that lower confidence was associated with larger action-updates (**A**). This relationship showed a good internal consistency (Spearman–Brown corrected Pearson correlations; **B**) and but low test–retest reliability (ICC; **C**). *Dots* in A show individual coefficients for T1 and T2 connected with thin lines, with their corresponding distribution to their right. The *bar plot* represents the mean model coefficient, and their *error bars* represent standard errors in **A**. Internal consistency in **B** for all measures is here displayed for T1. *Error bars* for internal consistency and test–retest reliability represent the estimates 95% confidence interval

We went on to further characterise these opposing patterns of action and confidence around the CPs. Specifically, we assessed how the participants’ action-update (i.e., the shortest distance between the previous bucket position and chosen bucket position on a given trial; cf. Methods) was related to their confidence rating on a given trial. Separate regression models for each participant, for both time points, showed that the size of the action-update was negatively predictive of confidence (T1: *β* = – 10.694, *SE* = 0.314, *p* < 0.001; T2: *β* = – 11.752, *SE* = 0.374, *p* < 0.001; cf. Fig. 3A). In other words, the larger the adjustment participants made to their bucket position, the less confident they were that it would catch the particle.

#### Psychometric properties

##### Internal consistency

To estimate the reliability of this negative relationship between action-update and confidence, we extracted the individual regression weights and computed their internal consistency and test–retest reliability (cf. Methods). We found that these regression weights had a good internal consistency at both time points (T1: *r*_*SB*_ = 0.759, 95% CI [0.709, 0.801]; T2: *r*_*SB*_ = 0.744, 95% CI [0.679, 0.798]; cf. Fig. 3B) which improved further when z-scoring the dependent variable (action-update; T1: r_*SB*_ = 0.808, 95% CI [0.767, 0.842]; T2: *r*_*SB*_ = 0.853, 95% CI [0.813, 0.886]). This indicates that the link between confidence and action-updates was stable within participants, within task runs.

##### Test–retest reliability

Examining the test–retest reliability of the confidence-action link, we found that the association showed a low reliability across time (ICC = 0.432, 95% CI [0.318, 0.533]; cf. Fig. 3C), which remained the similar when also *z*-scoring the dependent variable (ICC = 0.446, 95% CI [0.334, 0.546]). Thus, from one task administration to the other, the relationship between action-update and confidence was not stable, indicating that the association between the variables might not be the best measure to investigate inter-individual differences.

### Participants’ behaviour is linked to normative factors of a Bayesian learner

To link participants’ behaviour to further characteristics of the task, we assessed the link between their behaviour and variables derived from the quasi-optimal Bayesian learner (McGuire et al., 2014; Nassar et al., 2010, 2012, 2016, Nassar, Bruckner et al., 2019a). The Bayesian factors include the trial-wise change-point probability (CPP; measuring the model’s approximation of the probability that a CP has occurred) and relative uncertainty (RU; the model’s uncertainty about the mean of the Gaussian generating the particle landing location), in addition to participants’ own errors (i.e., PE^*h*^) and their accuracy (i.e., a binary regressor denoted as ‘Hit’). Subsequently, we computed the internal consistency and test–retest reliability scores for participants’ regression weights to examine how consistent the links between normative factors and behavioural measurements were within task runs and across time.

#### Action-update and the Bayesian learner

**Fig. 4:**
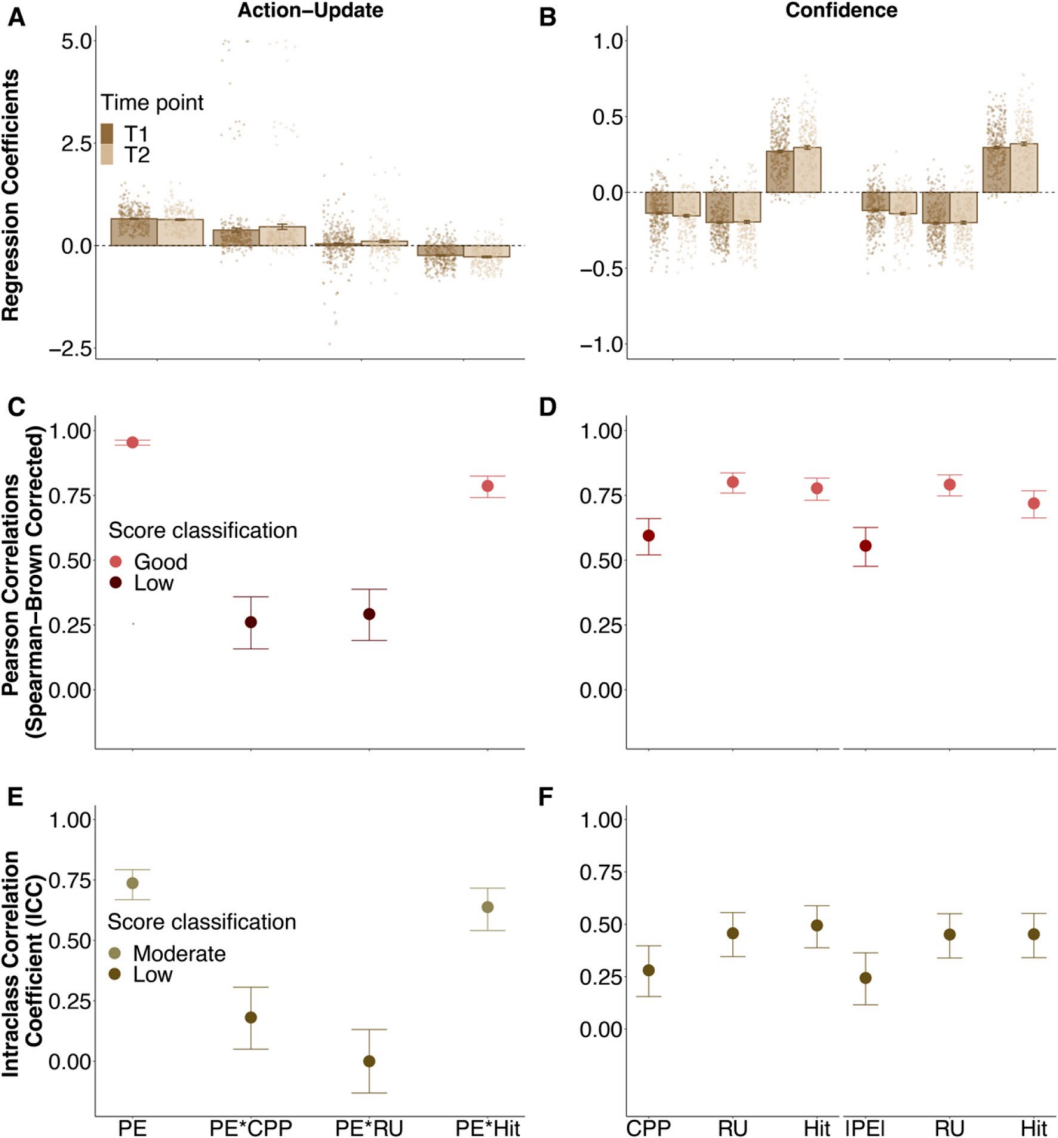
Bayesian model predictors and their relation to error-driven action-update and confidence ratings. A circular regression model predicted trial-wise action-updates based on PE^*h*^ (participants’ prediction error) and its interactions with the normatively-derived CPP (change-point probability) and RU (relative uncertainty) and Hit (accuracy on the previous trial). Action-update was positively linked to PE^h^ and its interaction with the normative factors. The interaction with Hit, participants' accuracy, was negatively predictive of action-update indicating that the effect of PE^h^ on action-update was attenuated when participants were accurate on the preceding trial. (**A**) Linear regression models predicted trial-wise confidence ratings on the basis of these Bayesian factors. Separate regression models were run for PE^*h*^ (left-side of each plot) and CPP (right-side of each plot). Normative regressors negatively predicted confidence while accuracy (Hit) predicted it positively (**B**). Investigating the robustness of the action-update associations, we saw that the internal consistency of its links to all normative interaction terms was low (Spearman–Brown corrected Pearson correlations) while the consistency of the beta weights for PE^*h*^ itself and its interaction term were good and moderate respectively **C**). In the confidence models, internal consistency of the beta coefficients for PE^*h*^ and CPP was moderate and the consistency of the RU and Hit regressor was good (Spearman–Brown corrected Pearson correlations; **D**). Test–retest reliability of all action-update regressors entailing a normative factor was low while PE^*h*^ and its interaction with Hit showed moderate reliability (as indicated by the ICC score;** E**). Test–retest reliability of the regression weights predicting confidence were all low (**F**). In **A**–**B**, individual coefficients of participants are represented by *circles*, while the *bar plot* represents the mean of the model coefficients, and their error bars represent standard errors. Internal consistency for all measures is here displayed for T1. *Error bars* for internal consistency and test–retest reliability represent the estimates 95% confidence interval.

We next investigated whether the behavioural updates were influenced by task characteristics captured by the Bayesian learner’s estimated CPP and RU as shown in previous research (e.g., Nassar et al., 2016, Nassar, Bruckner et al., 2019a; Seow & Gillan, 2020; Vaghi et al., 2017) and ran a circular regression model that predicted action-update based on participants’ PE^*h*^ and separate interaction terms between PE^*h*^ and all normative factors as well as participants’ own accuracy (i.e., Hit regressor) aiming to capture potential modulations of the error-driven learning. This analysis showed that action-update was positively linked to PE^*h*^ (T1:* β* = 0.654, *SE* = 0.014, *p* < 0.001; T2:* β* = 0.634, *SE* = 0.018, *p* < 0.001) and this link was positively modulated by CPP (T1:* β* = 0.378, *SE* = 0.045, *p* < 0.001; T2: *β* = 0.458, *SE* = 0.063, *p* < 0.001) and negatively modulated by the Hit regressor at both time points (T1: *β* = – 0.240, *SE* = 0.015, *p* < 0.001; T2: *β* = – 0.276, *SE* = 0.018, *p* < 0.001). RU positively modulated the link at the second time point only (T1: *β* = 0.041, *SE* = 0.025, *p* = 0.101; T2: *β*_*l*_ = 0.106, *SE* = 0.028, *p* < 0.001; cf. Fig. 4A).

Thus, the effect of participants’ own PE^*h*^ on their action-update was higher when the normatively estimated probability of a change-point and relative uncertainty (although only at the second time point) was high. In contrast, the effect of PE^*h*^ on action-update was lower when participants caught the particle on the preceding trial. These behavioural links to the quasi-optimal Bayesian learner replicate previous findings in the literature (Nassar, Bruckner et al., 2019a; Seow & Gillan, 2020; Vaghi et al., 2017).

##### Psychometric properties

*Internal consistency*

We then examined the internal consistency of the estimated regression coefficients and saw that it was low for all interaction terms measuring the modulation of error-driven learning by normative factors (T1: CPP: *r*_*SB*_ = 0.150, 95% CI [0.043, 0.254]; RU: *r*_*SB*_ = 0.198, 95% CI [0.092, 0.300]; T2: CPP: *r*_*SB*_ = 0.367, 95% CI [0.246, 0.476]; RU: *r*_*SB*_ = 0.259, 95% CI [0.131, 0.379]). The consistency of the interaction effect with accuracy (i.e., Hit; T1: *r*_*SB*_ = 0.795, 95% CI [0.751, 0.831]); T2: *r*_*SB*_ = 0.796, 95% CI [0.742, 0.840]) and of PE^*h*^ itself (T1: *r*_*SB*_ = 0.943, 95% CI [0.930, 0.954]; T2: PE^*h*^: *r*_*SB*_ = 0.925, 95% CI [0.904, 0.942]; cf. Fig. 4C), however, was good. This means, while the effect of participants’ own errors on action-update and its modulation by their own preceding accuracy was consistent throughout the task runs, the interaction with the normatively captured task factors was less stable.

*Test–retest reliability*

The stability of the regression weights over time showed a similar pattern. Test–retest reliability of the interaction effects with the normative predictors were low (CPP: ICC = 0.181, 95% CI [0.050, 0.306]; RU: ICC = 0.000, 95% CI [– 0.131, 0.131]). The reliability of the PE^*h*^ main effect and the beta weights of the interaction term with Hit was moderate (PE^*h*^: ICC = 0.737, 95% CI [0.668, 0.792]; Hit: ICC = 0.637, 95% CI [0.540, 0.716]; cf. Fig. 4E). Thus overall, the raw measures such as PE^*h*^ and Hit had a more stable link to action-update than any normatively captured characteristic of the task as described by the Bayesian learner. We also examined the psychometric properties of additional regression models that included the Bayesian factors separately yielding similar overall results that are described in the Supplemental Information.

#### Confidence and the Bayesian learner

We also investigated whether participants’ confidence ratings were linked to their PE^*h*^s and the factors derived from the Bayesian learner. We ran linear regression models (due to the above-reported co-linearity between the main effects PE^*h*^ and CPP; cf. Methods) that predicted confidence ratings on the basis of participants absolute PE^*h*^, all normative factors of interest (i.e., CPP and RU) and participants’ own accuracy (i.e., Hit regressor) on the preceding trial.

Replicating previous findings (Seow & Gillan, 2020; Vaghi et al., 2017), we saw that PE^*h*^ (T1:* β*_*PE*_^*h*^ = – 0.138, *SE* = 0.007, *p* < 0.001; T2:* β*_*PE*_^*h*^ = – 0.155, *SE* = 0.008, *p* < 0.001), CPP (T1: *β*_*CPP*_ = CPP = – 0.120, *SE* = 0.007, *p* < 0.001; T2: *β*_*CPP*_ = – 0.141, *SE* = 0.008, *p* < 0.001) and RU (T1: *β*_*PE-model*_ = – 0.199, *SE* = 0.007, *p* < 0.001;* β*_*CPP-model*_ = – 0.203, *SE* = 0.007, *p* < 0.001; T2:* β*_*PE-model*_ = – 0.195, *SE* = 0.010, *p* < 0.001;* β*_*CPP-model*_ = – 0.200, *SE* = 0.010, *p* < 0.001) were negatively predictive of confidence ratings. In contrast, participants’ accuracy on the preceding trial (i.e., Hit) predicted confidence positively (T1: *β*_*PE-model*_ = 0.270, *SE* = 0.008, *p* < 0.001; *β*_*CPP-model*_ = 0.295, *SE* = 0.008, *p* < 0.001; T2: *β*_*PE-model*_ = 0.295, *SE* = 0.011, *p* < 0.001; *β*_*CPP-model*_ = 0.320, *SE* = 0.011, *p* < 0.001; cf. Fig. 4B). Thus, participants’ confidence was linked to their own performance (as measured by PE^*h*^ and accuracy) as well as the normative factors approximating the probability of a change-point and relative uncertainty. We also ran an additional control model predicting confidence and combining the predictors PE^*h*^ and CPP in one model, which replicated the here reported findings (cf. Supplemental Information).

##### Psychometric properties

*Internal consistency*

We again examined the internal consistency of the estimated links and saw that coefficients of PE^*h*^ and CPP were moderately consistent (T1: PE^*h*^*: r*_*SB*_ = 0.556, 95% CI [0.476, 0.626]; CPP: *r*_*SB*_ = 0.595, 95% CI [0.520, 0.661]; T2: PE^*h*^: *r*_*SB*_ = 0.479, 95% CI [0.370, 0.575], CPP: *r*_*SB*_ = 0.528, 95% CI [0.426, 0.618]), while the internal consistency of the remaining predictors was good (T1: RU_*PE-model*_: *r*_*SB*_ = 0.792, 95% CI [0.748, 0.829]; RU_*CPP-model*_: *r*_*SB*_ = 0.801, 95% CI [0.759, 0.837]; Hit_*PE-model*_: *r*_*SB*_ = 0.719, 95% CI [0.663, 0.768]; Hit_*CPP-model*_: *r*_*SB*_ = 0.778, 95% CI [0.731, 0.817]; T2: RU_*PE-model*_: *r*_*SB*_ = 0.841, 95% CI [0.797, 0.876]; RU_*CPP-model*_: *r*_*SB*_ = 0.842, 95% CI [0.799, 0.877]; Hit_*PE-model*_: *r*_*SB*_ = 0.773, 95% CI [0.713, 0.821]; Hit_*CPP-model*_: *r*_*SB*_ = 0.818, 95% CI [0.769, 0.857]; cf. Fig. 4D). Thus, overall, all predictors had a relatively stable influence on confidence ratings throughout the task.

*Test–retest reliability*

Examining the test–retest reliability of the regression weights, we saw that it was low for all regressors (RU_*PE-model*_: ICC = 0.451, 95% CI [0.339, 0.550]; RU_*CPP-model*_: ICC = 0.457, 95% CI [0.346, 0.556]; Hit_*PE-model*_: ICC = 0.452, 95% CI [0.340, 0.552]; Hit_*CPP-model*_: ICC = 0.495, 95% CI [0.388, 0.588; CPP: ICC = 0.280, 95% CI [0.155, 0.397]; PE^*h*^: ICC = 0.244, 95% CI [0.116, 0.364]; cf. Fig. 4F). This indicates that associations between participants' confidence and the Bayesian model predictors may be less suitable for individual-difference approaches compared to other raw measures reported above.

### Associations between task measures and psychiatric dimension scores

Task reliability is crucial when studying inter-individual differences, as its absence can lead to inconsistent and spurious findings (Hedge et al., 2018). Previous research has utilised the predictive-inference task to explore behavioural differences in OCD patients and controls (Vaghi et al., 2017) as well as along psychiatric dimensions (Seow & Gillan, 2020) revealing contrasting driving factors (cf. Methods). Therefore, in our study, we sought to replicate these findings in our sample to further investigate their consistency. Contrary to other studies in the literature, we did not observe any link between any OC symptom scores and action-confidence coupling, meaning the symptom scores were not significantly associated with the beta values gained from the regression linking confidence and action-update (*r*_*S*_ = – 0.004, *p =* 0.95). Moreover, the scores also were not correlated with mean confidence (OC symptoms: *r*_*S*_ = – 0.025, *p =* 0.716) or action-update itself (*r*_*S*_ = 0.028, *p =* 0.678).

We also repeated the regression models predicting confidence and action-update based on normative factors, now additionally including the OC symptoms as a predictor. Previous literature had shown a decreased effect of CPP on confidence in individuals scoring high on the transdiagnostic compulsivity dimension (Seow & Gillan, 2020) and OCD patients (Vaghi et al., 2017). However, we did not find any significant correlation between the CPP beta weights of the regression model predicting confidence and OC symptoms (*r*_*S*_ =0.002, *p =* 0.972), nor the PE^*h*^ beta weights of the action-update model and OC symptom scores (*r*_*S*_ = 0.009, *p =* 0.896).

Finally, probing previous findings of an increased LR^*h*^ in OCD patients (Vaghi et al., 2017) in our general public sample, we investigated whether the OC symptom scores were linked to the mean LR^*h*^ but could not find any significant association (*r*_*S*_ = 0.011, *p =* 0.875; anxiety: *r*_*S*_ = 0.044, *p* = 0.518).

Given the data were collected during the COVID-19 pandemic, which has been seen to be associated with increased OC symptoms in the general public (e.g., Banerjee 2020; Loosen et al., 2021; Tanir et al. 2020), we conjectured that this general increase might have affected our replication attempt (we saw a trend-level increase in OCI-R scores compared to previous studies (Seow & Gillan, 2020), *t*(463.92) = 1.844, *p* = 0.066). When repeating the analysis using an OC symptom score that excluded all items that could have been influenced by the pandemic, our results did not change (cf. Supplemental Information). These findings remained true when using behavioural data from T1 instead (data not shown). Moreover, we conducted additional analyses considering psychiatric symptom scores that exhibit a significant overlap with OC symptoms in the general population (Gillan et al., 2016) and are commonly comorbid in patients with OCD (i.e., anxiety and depression; American Psychiatric Association, 2013). However, we also did not identify any statistically significant associations between these symptom scores and the measures investigated here (cf. Supplemental Information).

## Discussion

Across cognitive neuroscience and computational psychiatry, low reproducibility challenges the interpretability and generalisability of neurocognitive findings (Button et al., 2013; Poldrack et al., 2017; Szucs & Ioannidis, 2017). A particular challenge is the threat of poor psychometric properties of behavioural task measures, which can have considerable ramifications for both within- and between-subjects inference. In the present study, we have thus examined the psychometric properties, in particular the internal consistency and test–retest reliability, of a specific implementation of the widely used predictive-inference task (Bruckner et al., 2020; Jepma et al., 2016; Kao, Khambhati et al., 2020a; Kao, Lee et al., 2020b, 2020c; Krishnamurthy et al., 2017; McGuire et al., 2014; Nassar et al., 2012, 2016; Nassar, McGuire et al., 2019b; Nassar & Frank, 2016; Nassar & Troiani, 2020; Razmi & Nassar, 2020; Seow & Gillan, 2020; Vaghi et al., 2017) using a large-scale, re-test online sample.

When assessing the most commonly used behavioural measures, namely learning rate (LR^*h*^) and confidence before, at, and after environmental changes (i.e., CPs), we found a good overall internal consistency of both measures and show that current tasks could even be shortened while retaining sufficient consistency. We also found that test–retest reliability was mostly moderate for both measures, with the exception of a low reliability for LR^*h*^ at CPs due to low between-participant variability. This suggests that LR*h* is not recommended for use in between-participant variability designs, while the remaining measures appear well-suited for such designs. 

The good internal consistency of the two main ‘raw’ measures, LR^*h*^ and confidence, indicates that the task captures behavioural and belief adaptations to sudden changes well (Miller & Lovler, 2018). This stability is an important pre-requisite when considering e.g., the effect of experimental manipulations on task performance (Jepma et al., 2016) or associations within an individual such as the link between LR^*h*^ and confidence (Nassar et al., 2010). Moreover, the good reliability of the adaptation in LR^*h*^ from pre- to post-CPs underscores that LR^*h*^ appears to be a stable raw measure capturing changes in behaviour in a dynamic environment.

A similar picture emerges when investigating the test–retest reliability. The mostly moderate test–retest reliability of the ‘raw’ measures is particularly important for studies that have shown alterations in these measures in e.g., patients with schizophrenia (Nassar et al., 2021) or OCD (Vaghi et al., 2017) or those that use such metrics in combination with pharmacological or other interventions (e.g., Jepma et al. 2016). It is important to note that the ICC score used here to examine test–retest reliability is more conservative than other ICC scores (i.e., ICC scores for average measures; Koo & Li, 2016), which could also be argued for and would yield a good test–retest reliability. Our results thus validate many of the previous findings using these metrics and suggest that these measures can indeed be used with great confidence.

However, the psychometric properties of other common, but more complex, task measures were less consistent. Notably, metrics that are often used to assess the modulation of behavioural links (i.e., the link between participants’ errors and their actions) by complex task characteristics captured by a Bayesian learner were less stable and reliable. This cannot solely be attributed to differences in participants' use of a Bayesian strategy, as even if some individuals are not Bayes-optimal but consistent in their behaviour, the reliability of the measures would still be high. This main finding is, moreover, consistent across different approaches used in the literature to approximate a Bayesian learner. However, it should also be noted, that all approaches yielded the same overall results and main links between the normative factors and behaviour were replicated across studies with the exception of the link between people’s action-updates and the CPP-predictor, which was weaker in our sample than it has been in other studies (e.g., McGuire et al., 2014; Nassar, Bruckner et al 2019a, Nassar, McGuire et al. 2019b).

There are multiple factors that may have contributed to these findings. Firstly, these complex measures are interactions between multiple noisy task measures, which is known to lead to a larger overall measurement noise (Shahar et al., 2019; Waltmann et al., 2022). Secondly, we found that multiple model-derived predictors showed a high degree of co-linearity and thus directly affected how well the impact of these metrics could be measured when used in the same model (cf. Supplemental Information for an assessment across different modelling approaches).

This co-linearity is to some extent inherent in the Bayesian model. However, if task settings, such as the hazard rate and the spread of the particle-generating Gaussian distribution (i.e., SD) were varied across the experiment (e.g., Kao, Khambhati et al. 2020a; Nassar et al. 2010; Seow and Gillan 2020), this apparent co-linearity could be reduced, which may help improve the psychometric properties of these measures. Moreover, our task design of choice might limit the ability to differentiate between changepoint-driven learning rate adjustments and the effects of successful predictions ('Hit'). This overlap, illustrated in Supplemental Fig. 7, could be influenced by the model's assumed hazard rate. For instance, varying the hazard rate, as implemented in some previous research (e.g., Kao, Khambhati et al., 2020a; Nassar et al., 2010; Seow and Gillan, 2020), could help to isolate these effects. Such variations in task parameters might also elicit distinct behavioural strategies, which should be considered when selecting a task paradigm (Tavoni et al., 2022) and explain why the link between CPP and behavioural updates appeared weaker in our sample. This highlights the importance of careful task design and parameter selection and definition. Future research may shed more light on this by examining different task adaptations (e.g., with varying hazard rates and/or standard deviations of the generative distribution) and exploring their psychometric properties.

The lower psychometric properties of the links between behaviour and the Bayesian learner may also be the reason why we did not find any association with OC symptoms. However, we also did not observe any links to the simpler task measures, such as mean confidence, as observed previously (Seow & Gillan, 2020; Vaghi et al., 2017). This may also be due to other differences between the studies such as the definition of the psychiatric variable (i.e., questionnaire score versus clinical diagnosis versus cross-questionnaire factors), the samples' cultural background (i.e., British versus American samples), recruiting sources or other potentially unaccounted-for variables.

It is important to highlight that the interval between test and re-test can substantially affect reliability estimates. Our time interval (~ 3 months) between testing sessions aligns with the timescale of many studies on behavioural change. While this underpins the robustness of the reliable measures, it should also be noted that a shorter interval are likely to increase these estimates (Calamia et al., 2013; McCaffrey & Westervelt, 1995). Moreover, the overlapping confidence intervals of different psychometric scores reported here, particularly between raw and complex variables, suggest a need to re-evaluate the clear distinctions within pre-established categories). In addition, future studies should examine whether a lab-based version or one with different settings may hold different psychometric properties. However, given that they are believed to measure the same constructs, a large divergence between task types would be worrying. Lastly, as mentioned above, the ongoing COVID-19 pandemic might have altered behavioural as well as psychiatric measures. While we controlled for pandemic-related items in our psychiatric questionnaires, behavioural measures such as e.g., confidence might have been affected in subtle ways we could not account for.

In conclusion, we show that the main and simpler measures of this predictive-inference task consistently and reliably capture belief and behavioural adaptations before and after environmental changes, making them suitable for studies investigating individual differences. We also point out that the complex links between task variables and model predictions were of lesser psychometric quality. As a result, raw behavioural measures, such as the learning rate adaptations, appear to be particularly suited for investigating behavioural changes in dynamic environments. Our findings highlight the importance of a careful task design and thorough assessment of the task measures’ psychometric quality as such properties can differ within the same task.

### Supplementary Information

Below is the link to the electronic supplementary material.

Supplementary file1 (DOCX 3202 KB)

## Data Availability

Code for data analysis of this study is available from a dedicated GitHub repository (https://github.com/amloosen/A-Psychometric-Investigation-of-the-Predictive-Inference-Task) and fully-anonymised data is available at https://osf.io/hakq9/.

## Declarations

### Preregistration

This study was not preregistered.
