# Supplementary material for: Consistency within change: Evaluating the psychometric properties of a widely used predictive-inference task
Source: Behav Res Methods. 2024 Jun 6;56(7):7410–26. doi: 10.3758/s13428-024-02427-y (PMC11362202; doi:10.3758/s13428-024-02427-y)
Supplement: Supplementary file 1 — Supplementary file1 (DOCX 3202 KB) [file 13428_2024_2427_MOESM1_ESM.docx]

Supplemental Information for

Consistency within change: Evaluating the psychometric properties of a widely-used predictive-inference task

Alisa M. Loosen^1,2^, Tricia X.F. Seow^1,2^ & Tobias U. Hauser^1,2^

^1^Max Planck UCL Centre for Computational Psychiatry and Ageing Research

^2^Wellcome Centre for Human Neuroimaging, University College London

Supplemental Methods

# Exclusion criteria

At both time points (time point 1, T1; time point 2, T2), we excluded participants because of failed attention checks (T1: *N*=19; T2: *N*=14) or inconsistent game performance. Exclusion criteria for game performance were aligned with those used by Seow and Gillan (2020) and as follows:

1. The confidence rating was left unchanged, thus equal to the default rating, in more than 60% of the trials (T1: *N*=14; T2: *N*=4).
2. The task was reset more than five times, whereby the reset was prompted if participants left as the default confidence rating for more than 70% of the first 50 trials. This was not the case for any of our participants.
3. If across trials, the default confidence rating correlated more than 0.5 with the confidence rating logged in by the participant (T1: *N*=17; T2: *N*=17).
4. If the mean confidence on trials that were preceded by a correct trial (i.e. caught the particle on the trial before) was lower than the mean confidence on trials that were preceded by an incorrect trial (i.e. did not catch the particle on the trial before; T1: *N*=19; T2: *N*=6).

We additionally excluded participants who used the same confidence rating more than 90% of the time (T1: *N*=2; T2: *N*=0) to ensure usage of the full confidence scale.

# Normative parameters of the reduced quasi-optimal Bayesian learner

To capture task characteristics thought to influence people’s behaviour and confidence in changing (task) environments, we fitted the reduced quasi-optimal Bayesian learner that had been used by past studies using this task (e.g. McGuire et al. 2014; Nassar, Bruckner, and Frank 2019; Seow and Gillan 2020; Vaghi et al. 2017) to the trial-wise task data. Normative parameters used in the main analyses are marked in bold.

This Bayesian learner updates its belief about the landing position distribution (i.e. point estimation of the mean of the Gaussian distribution from which the particle locations were sampled) using a delta-rule (equation 1).

| $B_{t+1}=B_{t}+{\mathrm{LR}^{b}}_{t}\times{\mathrm{PE}^{h}}_{t}$ | (1) |
| --- | --- |

The learner thereby weights the participants' prediction error (rather than the learner's prediction error (cf. Alternative approximation of the Bayesian learner) by the LR*^b^*_,_ thus, determining how much the encountered particle landing position will influence the learner’s belief on the next trial. This new conceptualization of the Bayesian learner ensures, that the learner’s estimates are based on the participants' actual experience (Nassar et al., 2016). The LR*^b^* itself (equation 2) is determined by the change-point probability (i.e. Ω or **CPP**) and the learner's relative uncertainty (i.e. τ or $\mathbf{RU}$).

| ${\mathrm{LR}^{b}}_{t}=\Omega_{t}+(1-\Omega_{t})RU$ | (2) |
| --- | --- |

The **CPP** captures how likely it is that the mean of the generative distribution determining the particle location has shifted (equation 3). **RU** captures the model’s uncertainty caused by an inaccurate estimation of the mean of the generative distribution or more specifically the relative uncertainty in the belief about the mean of the generative distribution determining the particle landing location (equation 5).

Unpacking the parameters further, **CPP** (equation 3) is more precisely described as the relative likelihood that the particle falling location is sampled from a new generative distribution, i.e. a change-point occurred (the distribution mean is determined by a uniform distribution U over all 360 possible locations; numerator of equation 3), or the falling location is again drawn from the same Gaussian (*N*) as the particle before, i.e. the Gaussian centred around B*_t_* (second term in the denominator of equation 3). Both scenarios (mean of the generative distribution has changed or not) are additionally influenced by the hazard rate H, which has been pre-determined during task development (H=0.125) and is the probability that the mean of the distribution has changed.

| $\Omega_{t}=\frac{U\left( X_{t} \vert1,360 \right)H}{U\left( X_{t} \vert1,360 \right)H+N\left( X_{t} \vert B_{t},\sigma_{t}^{2} \right)(1-H)}$ | (3) |
| --- | --- |

The term $\sigma_{t}^{2}$ (equation 4) thereby represents the estimated variance of the predictive distribution of the particle falling locations. This, in turn, is determined by the variance of the overall generative Gaussian distribution $\sigma_{N}^{2}$ modulated by RU. This means, the larger the MC, the smaller the estimated variance of the predictive distribution (or the closer to $\sigma_{N}^{2}$). As model confidence increases with trials after the change-points, the certainty about the particle falling location increases. Since our task entailed a fixed hazard rate and $\sigma_{N}^{2},$CPP was solely driven by a mismatch between the newest particle landing location and prior expectations.

| $\sigma_{t}^{2}=\sigma_{N}^{2}/\tau_{t}$ | (4) |
| --- | --- |

In contrast to all other parameters, RU (equation 5) is computed at the end of each trial (*t*) for the subsequent trial (*t+1*). The numerator contains a weighted average of the variance of the overall generative Gaussian distribution ($\sigma_{N}^{2}$) (first term) and $\sigma_{N}^{2}$ conditional on no change-point (second term). It also includes a term capturing the variance due to the difference in means of these two conditional distributions (third term). The denominator is almost identical to the numerator but entails an additional term capturing uncertainty due to noise ($\sigma_{N}^{2}$).

| $\tau_{t+1}=\frac{\Omega_{t}\sigma_{N}^{2}+\left( 1-\Omega_{t} \right)\sigma_{N}^{2}{\tau_{t}+\Omega}_{t}(1-\Omega_{t}){(\delta_{t}(1-\tau_{t}))}^{2}}{\Omega_{t}\sigma_{N}^{2}+\left( 1-\Omega_{t} \right)\sigma_{N}^{2}{\tau_{t}+\Omega}_{t}(1-\Omega_{t}){(\delta_{t}(1-\tau_{t}))}^{2}+\sigma_{N}^{2}}$ | (5) |
| --- | --- |

As described in the manuscript, trial-wise estimates of **CPP** and **RU** were used to capture how surprise and uncertainty due to changes in the task influenced participants’ actions and confidence.

# Alternative approximation of task variables and the Bayesian learner

To probe further analyses used in the literature (Seow & Gillan, 2020; Vaghi et al., 2017), we also implemented regressions that conceptualized action-update, PE*^h^* and LR*^h^* as the shortest, total distance instead of a circular distance. We re-ran our main analyses with these alternative variables. Moreover, we also approximated the Bayesian learner according to an approach in the literature (McGuire et al., 2014; Nassar et al., 2010; Seow & Gillan, 2020; Vaghi et al., 2017) that had preceded the one adapted in the main manuscript (Nassar et al., 2016, 2019, 2021). Specifically, we re-computed the learner's normative factors on the basis of its own PE*^b^* instead of the PE*^h^* made by the participants (McGuire et al., 2014; Nassar et al., 2010; Seow & Gillan, 2020; Vaghi et al., 2017). The PE*^b^* (equation 6) captures the distance between the belief of the Bayesian learner where the particle landing position is (i.e. B*_t_*) and the location where the particle ended up falling to (i.e. X*_t_*). Moreover, as done in other previous research using the circular version of this task (Seow & Gillan, 2020; Vaghi et al., 2017), in alternative analyses reported here, we conceptualized this distance as the shortest, linear total distance between the two positions on the circle rather than the circular distance.

| ${\mathrm{PE}^{b}}_{t}={X_{t}-B}_{t}$ | (6) |
| --- | --- |

In these additional analyses, we also adapted the regressor RU by multiplying it with the inverse of CPP (i.e. $\left( 1-\Omega_{t} \right)*RU)$ following the approach taken by Seow and Gillan (2020) and Vaghi and colleagues (2017), which also corresponds to the final term of the LR*^b^.*

# Alternative regression models

To estimate the reliability of alternative analysis approaches used in the field, we implemented alternative regression models and estimated the resulting parameters' psychometric properties. First of all, we repeated the regression models predicting action-update but ran separate models for PE*^h^* and CPP accounting for their high correlation (cf. Methods).

Secondly, we implemented an alternative analysis approach followed by Vaghi et al. (2017) and Seow and Gillan (2020), which relies on the shortest linear distance on the circle when computing action-update and PEs. Following a summary statistics approach, we used linear regression models to predict action-update and confidence on the basis of all linear versions of behavioural and Bayesian variables (cf. above). In addition we also ran mixed-effects models implemented by Seow and Gillan (2020) with these alternative variables as well as demographic fixed-effect covariates (i.e. IQ, gender and age). Covariates were z-scored across participants, while normative factors, confidence and action-update were z-scored within participants.

It should be noted that these mixed-models did not converge using our dataset. In an attempt to achieve convergence, we followed a step-by-step-approach lined out in the literature (Bates et al., 2018), simplifying and adapting the models. This entailed a simplification of the models and the change of the optimizer (i.e. *bobyqa*). However, further steps such as the removal of correlations between random effects via orthogonalization, were not feasible as they would have made a valid reliability analysis of the mixed-model weights impossible.

We computed the mixed-effects models using the *lme4* package (Bates et al., 2015) in R. We used the same predictors as in the summary statistics approach described above.

In the syntax of the *lme4* package (Bates et al., 2015, p. 4), the model was specified as follows:

*Action-Update ~ PE^b^ + CPP + RU + Hit + Age + IQ + Gender + (1 + PE^b^ + CPP + RU* | *Participant)*

When we subsequently investigated whether psychiatric questionnaire (total) scores were associated with the normative factors, we ran separate regression models for each of them. The psychiatric variables (i.e. OC symptoms, anxiety, and depression) were all z-scored across participants and included as between-subject predictors as follows:

*Action-Update ~ (PE^b^ + CPP + RU+ Hit) * Psychiatric Variable + Age + IQ + Gender + (1 + PE^b^ + CPP + RU* | *Participant)*

All normative factor regressors used to predict action-update were implemented as an interaction effect with PE*^b^*.

Supplemental Results

To illustrate the distribution of learning rates among participants, we created histograms after truncating the data to values between 0 and 1. One histogram shows aggregated learning rates across all participants (cf. Supplemental Figure 1A). Additionally, we generated histograms for two selected participants (cf. Supplemental Figure 1B) to provide concrete examples, specifically for the participant with the highest mean learning rate (LR=0.844) and the one with the lowest mean learning rate (LR=0.155).


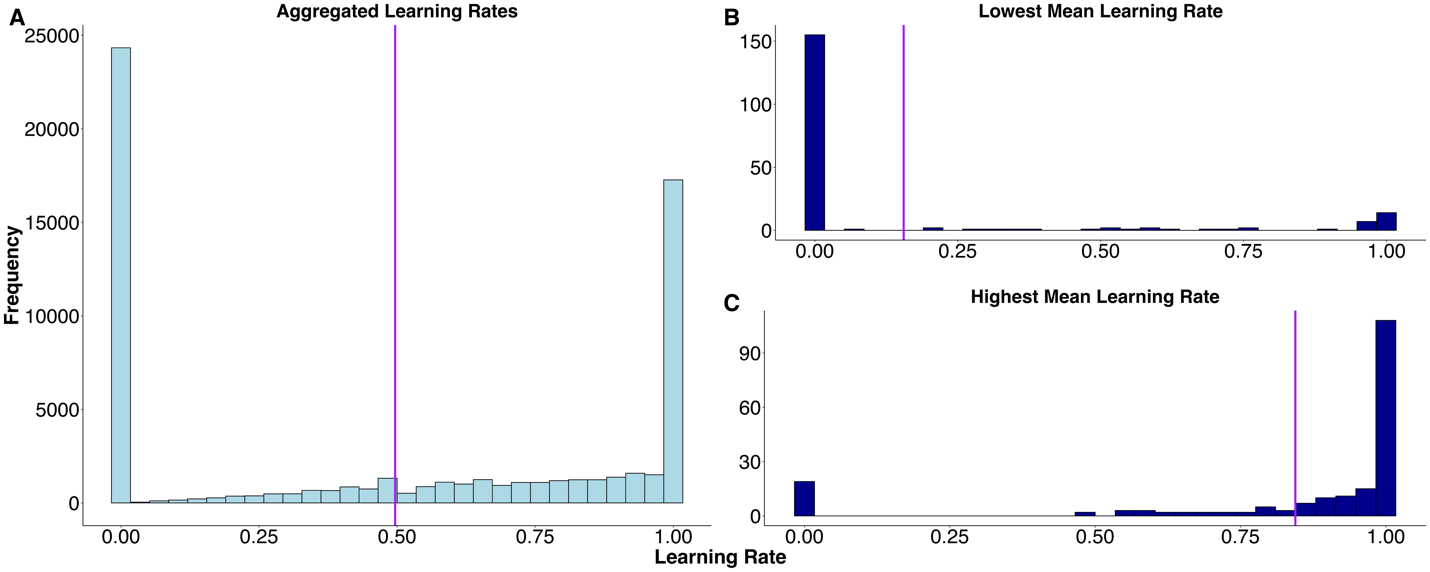


*Supplemental Figure 1.* Histograms of Learning Rates. Aggregated histogram displaying the distribution of learning rates across all participants (**A**). Individual histograms for two representative participants, one with the highest mean learning rate (LR=2.960) and another with the lowest mean learning rate (LR=0.185), illustrating the variability in learning rates within the dataset (**B**). The vertical purple lines represent the mean value of the data in the respective subplot.

# Internal consistency and test-retest reliability of learning rate and confidence

**Supplemental Table 1**

*Internal Consistency and Test–Retest Reliability of Behavioural Measures from the Predictive-Inference Task*

|  |  | Learning Rate (LR*^h^*) | | | |  | | Confidence | | | | |  |
| --- | --- | --- | --- | --- | --- | --- | --- | --- | --- | --- | --- | --- | --- |
|  |  | Internal Consistency (*r_SB_*) | |  | Test-retest reliability (*ICC*) | |  | | Internal Consistency (*r_SB_*) | |  | Test-retest reliability (*ICC*) | |
| Trial |  | T1 | T2 |  |  | |  | | T1 | T2 |  |  | |
| -4 |  | 0.768 [0.720 0.809] | 0.838 [0.794 0.874] |  | 0.613 [0.523, 0.690] | |  | | 0.928 [0.911, 0.941] | 0.914 [0.890, 0.934] |  | 0.531 [0.408, 0.631] | |
| -3 |  | 0.744 [0.692 0.789] | 0.776 [0.718, 0.8242] |  | 0.580 [0.485, 0.662] | |  | | 0.922 [0.904, 0.937] | 0.945 [0.929, 0.959] |  | 0.505 [0.396, 0.600] | |
| -2 |  | 0.804 [0.762 0.839] | 0.833 [0.787, 0.869] |  | 0.678 [0.600, 0.744] | |  | | 0.946, [0.933, 0.956] | 0.930 [0.909, 0.946] |  | 0.507 [0.394, 0.603] | |
| -1 |  | 0.766 [0.718 0.808] | 0.829 [0.782, 0.866] |  | 0.595 [0.502, 0.674] | |  | | 0.934 [0.919, 0.947] | 0.938 [0.920, 0.952] |  | 0.480 [0.365, 0.579] | |
| 0 (at CP) |  | 0.699 [0.639 0.750] | 0.750 [0.685, 0.803] |  | 0.156 [0.026, 0.282] | |  | | 0.921 [0.903, 0.936] | 0.931 [0.911, 0.947] |  | 0.560 [0.451, 0.651] | |
| 1 |  | 0.624 [0.553 0.686] | 0.684 [0.606, 0.748] |  | 0.545 [0.445, 0.632] | |  | | 0.938 [0.924, 0.950] | 0.948 [0.933, 0.960] |  | 0.492 [0.379, 0.589] | |
| 2 |  | 0.775 [0.728 0.815] | 0.798 [0.744, 0.842] |  | 0.575 [0.479, 0.657] | |  | | 0.924 [0.906, 0.938] | 0.913 [0.889, 0.933] |  | 0.505 [0.392, 0.601] | |
| 3 |  | 0.737 [0.684 0.783] | 0.815 [0.765, 0.855] |  | 0.585 [0.486, 0.669] | |  | | 0.919 [0.900, 0.934] | 0.944 [0.927, 0.956] |  | 0.524 [0.412, 0.619] | |
| 4 |  | 0.759 [0.709 0.801] | 0.779 [0.721, 0.826] |  | 0.650 [0.562, 0.723] | |  | | 0.930 [0.914, 0.944] | 0.922 [0.899, 0.939] |  | 0.482 [0.357, 0.587] | |
| Entire task |  |  |  |  | 0.570 [0.474, 0.653] | |  | |  |  |  | 0.541 [0.424, 0.638] | |

*Note.* Trials refer to four trials before (-4), at (0) to four trials after (4) a change point (CP) and for completeness, we also computed the test-retest reliability over all trials of the task. *ICC*=intraclass correlation of absolute agreement; *r_SB_*=Spearman-Brown corrected correlations; 95% confidence intervals are specified in square brackets.

***
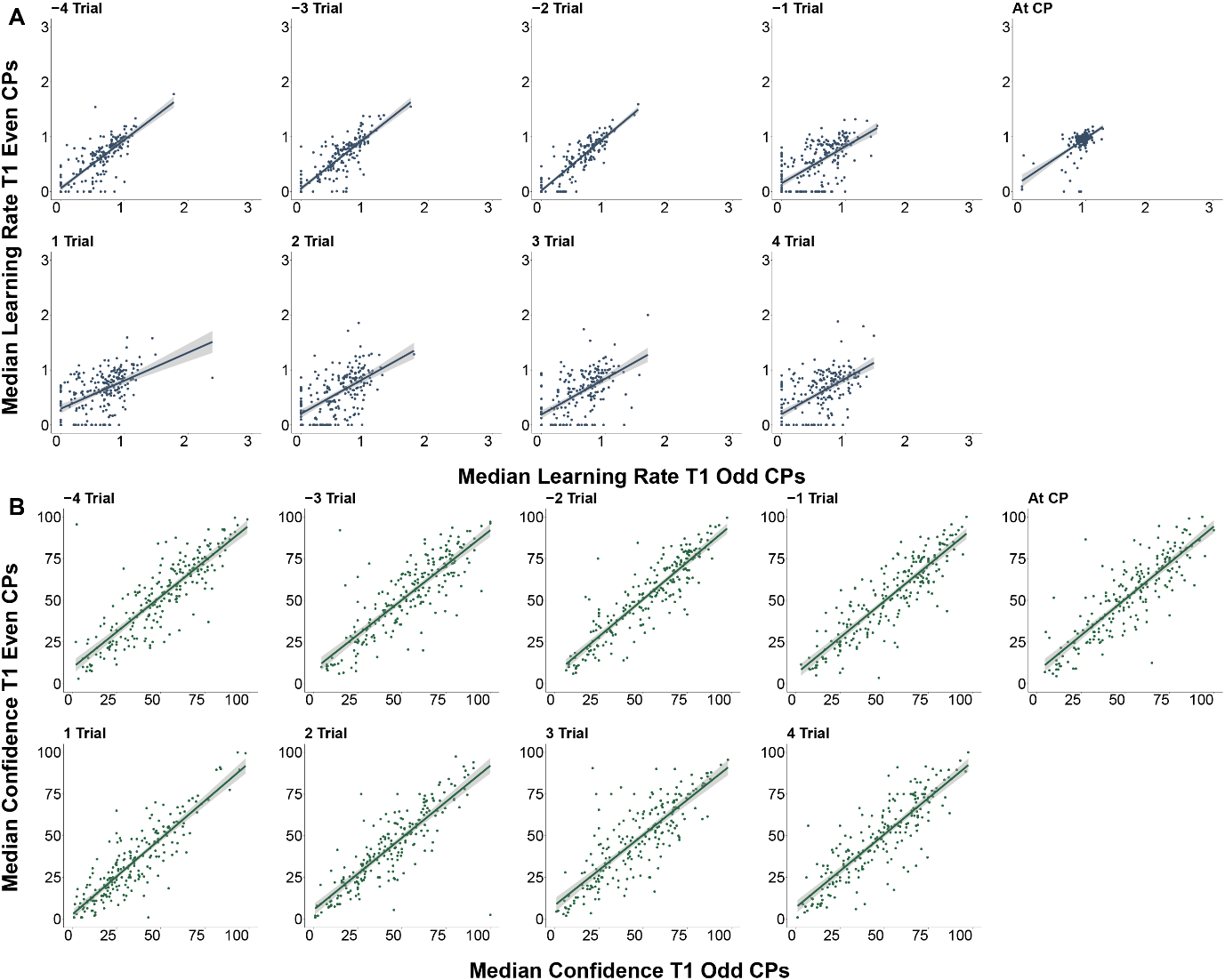
***

Supplemental Figure 2. Internal consistency of median learning rate (**A**) and median confidence (**B**) at time point 1. Represented is the consistency of median values between odd change-points (CP; x-axes) and even CPs (y-axis) at trials ranging from 4 before (-4) to 4 after (4) a CP. Data at time point 2 showed a very similar pattern (cf. Supplemental Table 1).


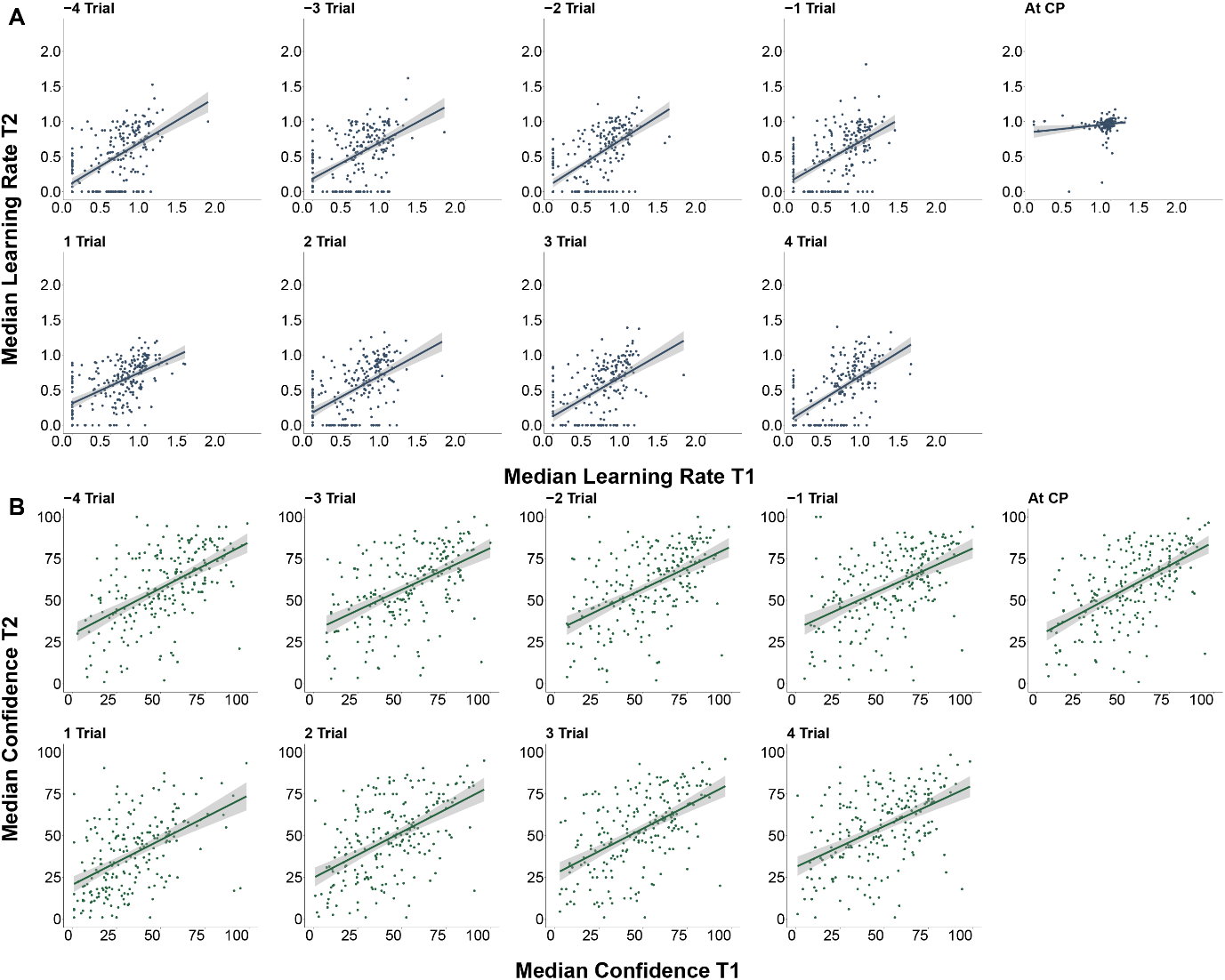


Supplemental Figure 3. Test-retest reliability of median learning rate (**A**) and median confidence (**B**). Represented is the agreement of median values between time point 1 (T1; x-axes) and time point 2 (T2; y-axis) at trials ranging from 4 before (-4) to 4 after (4) a CP.

# The effect of task length on stability

To assess whether the task could potentially be shortened in future studies, we additionally investigated the internal consistency and test-retest reliability of LR*^h^* in shorter task-versions with 6 to 24 CPs (full task). To do so, we used the larger dataset from T1 (*N*=330) and computed psychometric scores for all CP counts separately, ranging from 6 CPs (to have a minimum of 3 odd and 3 even CPs capturing internal consistency) until the maximum in our task version, i.e. 24 CPs, was reached.

This showed, that with the here used hazard rate of 0.125, the LR*^h^* at trials before the CPs reached good internal stability (r*_SB_*=0.70) after ~22 CPs and at trials after and at the CPs the r*_SB_* score of the LR*^h^* reached this threshold after ~23rd CPs (cf. Supplemental Figure 4A-B). Only the internal consistency of the median LR*^h^* at the first trial after the CPs stayed moderate (r*_SB_*=0.50) from ~10 CPs onwards. Test-retest reliability of the LR*^h^* at most trials before the CPs reached a moderate reliability after ~18 CPs while it took until ~22CPs to reach moderate test-retest reliability after the CPs. Test-retest reliability of the LR*^h^* at the CP itself never reached a moderate level, which was explained by a low between-participant variance (cf. Supplemental Figure 3A). Overall, this shows that LR*^h^* investigations should entail at least as many CP-counts as implemented here.

The same analyses for confidence showed that it reached a good internal stability at all trials after only 7 CPs (cf. Supplemental Figure 4E-F). While its test-retest reliability took until the end of the task (i.e. ~24 CPs) to reach moderate test-retest reliability for the majority of trial categories (cf. Supplemental Figure 4G-H). None of these scores ever reached a good reliability level.


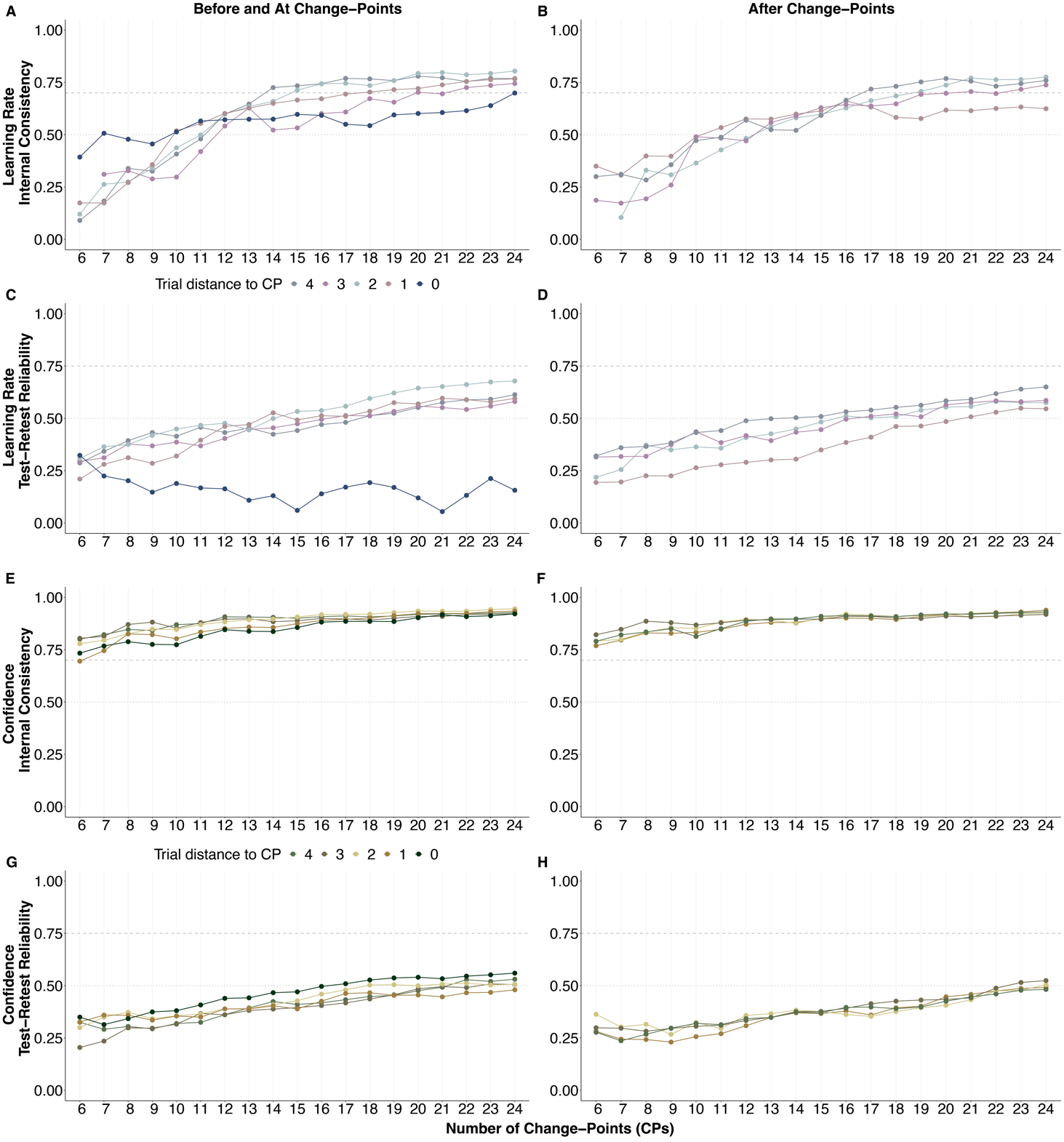


*Supplemental Figure 4.* Psychometric properties of human learning rate and human confidence with increasing change-points (CPs). The internal consistency (Spearman-Brown corrected Pearson correlations) of the learning rate (LR*^h^*) at T1 (*N*=330) stabilized after ~12 CPs and the measurement reached a good internal consistency at the majority of investigated trials at ~23 CPs (**B**). Test-retest reliability of the LR*^h^* as captured by the intraclass correlation (*ICC*) reached a moderate level at the majority of trials after ~ 18 CPs but stayed low at the CP itself (**C-D**). Internal consistency of confidence already stabilized on a good level at ~7 CPs for all trials (**E-F**). Test-retest reliability of confidence reached a moderate level only towards the end of the task (~24CPs; **G-H**). Horizontal dashed lines represent the cut-off score for good (*r_SB_*=0.70; ICC=0.75) and dotted lines represent the moderate cut-off score (*r_SB_*=0.50; ICC=0.50).

# Linear absolute approximation of the learning rate

An alternative approach to compute action-update and PE*^h^* and therefore also LR*^h^* in this task is to use the shortest absolute distance between two positions on the circle (cf. Supplemental Methods). We also computed LR*^h^* according to this approach and showed that the behavioural patterns reported in the main manuscript did not change (cf. Supplemental Figure 5A).

## Internal Consistency

We again investigated how consistent the median LR*^h^* was at each time point and found that the internal consistency was good at all trials and time points (*r_SB_*$\geq0.716$; cf. Supplemental Figure 5B) similar to the results of the circular version of LR*^h^* reported in the main manuscripts.

## Test-Retest Reliability

We again estimated the test-retest reliability of the average LR*^h^* scores and replicated the main findings in the main manuscript, showing that LR*^h^* was predominantly moderate (*ICC*$\geq$0.590; cf. Supplemental Figure 5C).The test-retest reliability of LR*^h^* at the CP itself was again low due to low inter-individual differences (*ICC*=0.075, 95% *CI* [-0.057, 0.205]).


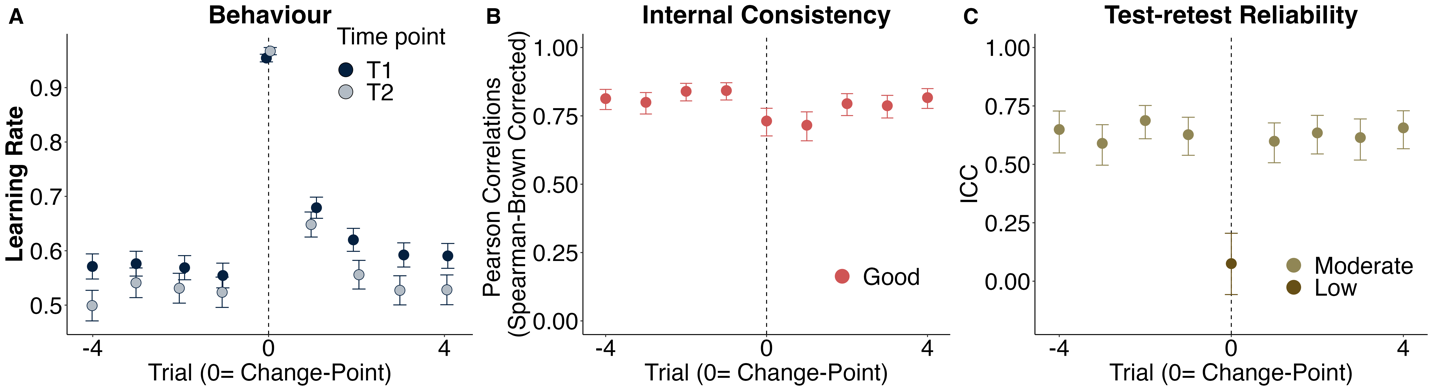


Supplemental Figure 5. Behavioural and psychometric properties of the non-circular learning rate. Participants’ learning rates (LR^h^) at time point 1 (T1; N_T1_=330) and 2 (T2; N_T2_=219) were again highest at the change-point (CPs; vertical dashed line) and decreased afterwards back to their pre-CP levels (**A**). Internal consistency (Spearman-Brown corrected Pearson correlations) for the median total LR^h^ was good at all investigated trials (here displayed for T1; **B**). Test-retest reliability measured by ICC-scores between median LR^h^s at T1 and T2 were mostly moderate before and after the CP but low at the CP itself (**C**). Error bars represent standard errors in A. Error bars for **B-C** represent the estimates 95% confidence interval.

# Raw behavioural measures and factors of the Bayesian learner throughout the task

Since RU, derived from the Bayesian Learner, was not as strongly associated with action-updates in our sample as it has been in the past (e.g. McGuire et al., 2014; Nassar et al., 2019), we inspected the trajectory of people’s behaviour and RU and CPP across a task run. We plotted participants' bucket updates, their accuracy, and the particle landing position, as well as the development of CPP and RU from one trial to the next for a participant whose action-update was highly linked to RU and for one whose update showed a weak link to RU (cf. Supplemental Figure 6). This visual inspection showed that both participants displayed behaviour in line with previous studies using this task (e.g. McGuire et al., 2014; Nassar et al., 2019). However, the regression results reported in the main manuscript suggest that our sample might have relied more on simpler indicators (e.g. their own accuracy) and less on complex approximations when playing the task (e.g. approximations of CPP or RU).

*
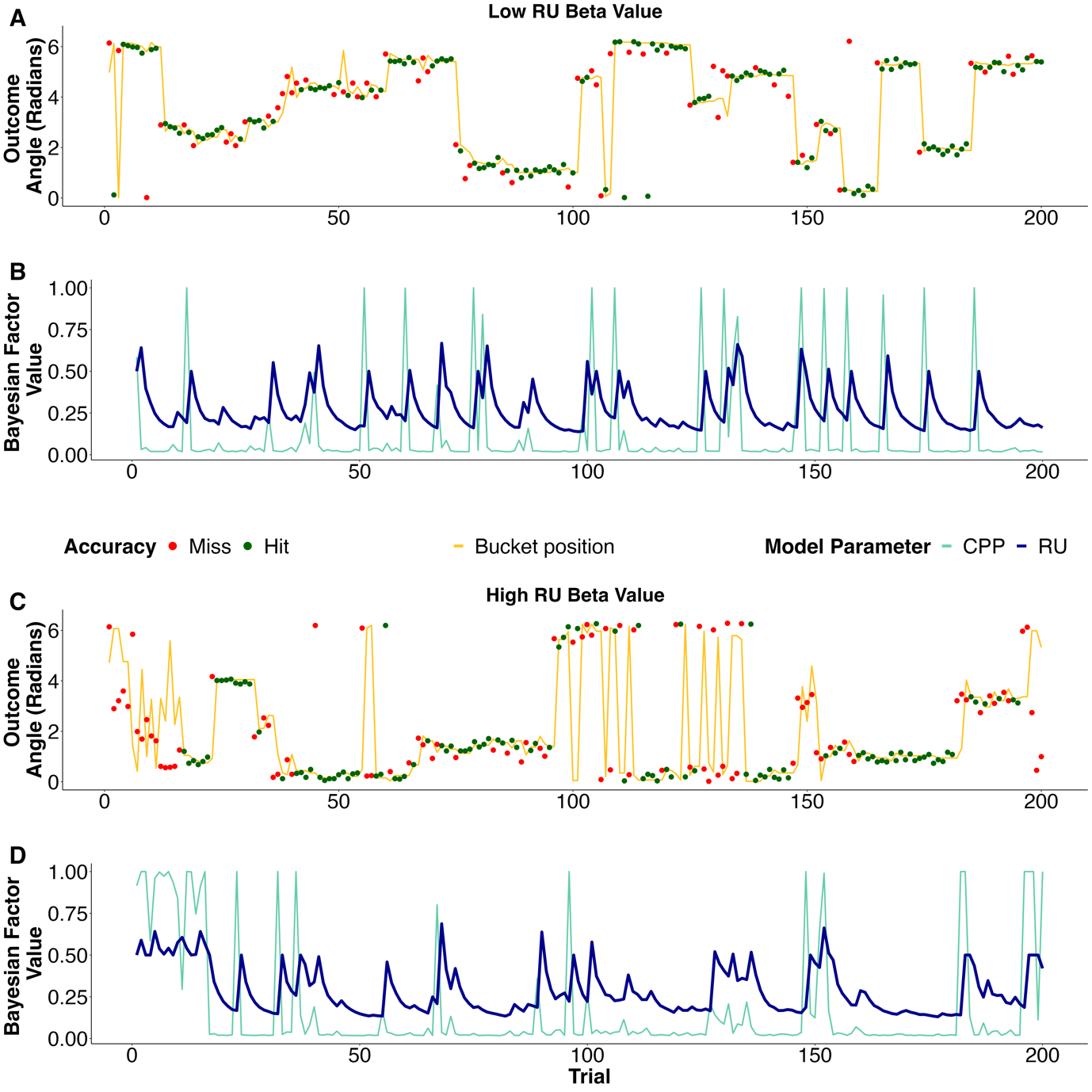
*

*Supplemental Figure 6*. Behavioural and Bayesian factor dynamics are displayed for one participant, in whom the relative uncertainty (RU) as approximated by the Bayesian learner was not (**A-B**) and one for whom it was highly predictive (**C-D**) of their action update. Points indicate the positions where particles landed on the circle, with their colour representing the accuracy of the corresponding trial (red for when the particle was not caught, and green for when it was caught). The yellow line denotes the bucket's position in both panels (**A&C**). Panels B and D illustrate the trajectory of the Bayesian factors change-point probability (CPP) and relative uncertainty (RU) for the respective participants (**B&D**).

Additional visual inspections of our data, relating it to previous publications using the predictive-inference task (McGuire et al., 2014; Nassar et al., 2019), we examined the relationship between participants signed action-update and PE, while relating it to the accuracy (Hit-range/ PE-range in which the particle was still caught) and CPP Bayesian learner. To illustrate these connections, we have selected two exemplary participants—one with a high average learning rate and another with a low average learning rate. The resulting plots, as shown in Supplemental Figure 7 demonstrate a positive linear relationship between action-update and PE and that lower CPP-values are associated with reduced PEs and subsequent action-updates, while higher CPP-values display the inverse relationship. Moreover, while the participant with a higher LR (Supplemental Figure 7A) seems to scale their action-updates according to PE size even when they did not catch the particle (i.e. outside of the Hit-range), the participant with the lower LR seems to inform their action-update largely by the binary feedback of whether they caught the particle (i.e. Hit; Supplemental Figure 7B; similar to data patterns observed in Nassar et al., 2019).


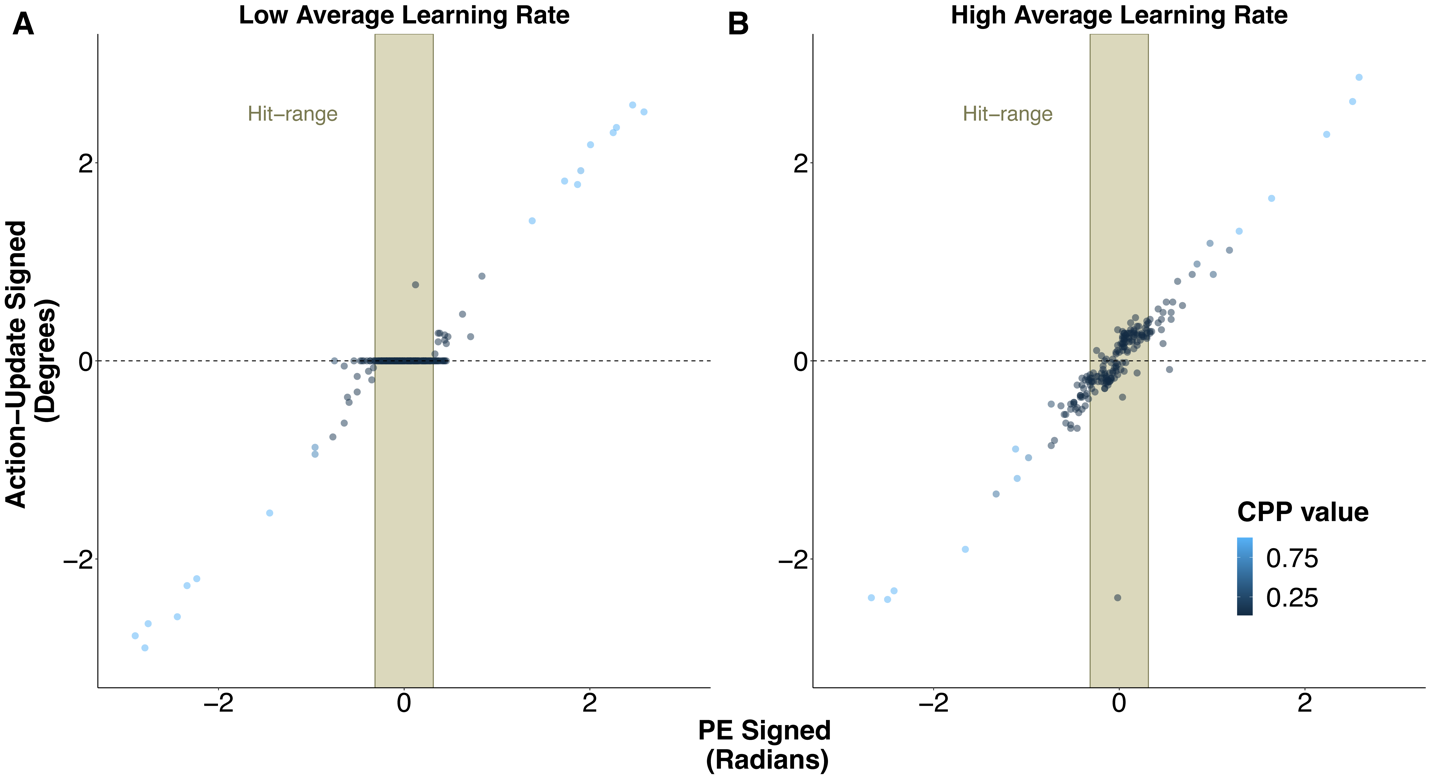


*Supplemental Figure 7*. A positive linear relationship between participants signed action-updates and prediction errors (PE) as exemplified by a participant with a low (**A**) and a participant with a high (**B**) mean learning rate. Data-points are marked according to the CPP-value associated with the specific trial showing that lower CPP-values (darker blue) are associated with smaller PEs and action-updates and higher CPP-values (lighter blue) with larger PEs and action-updates. The green column (i.e. Hit-range) indicates the range in which the participants successfully caught the particle.

# Alternative regression and correlation approaches

## Separate regression models predicting the circular action-update

We implemented separate circular regression models for a control analysis accounting for the discussed high correlation between PE*^h^* and CPP. We wanted to investigate whether separate models for the two variables regressors (i.e, PE*^h^* and PE*^h^**CPP) would yield the same behavioural and psychometric results as the combined model in our main analysis.

Indeed action-update was again positively linked to PE*^h^* (T1: *β*=0.680, *SE*=0.012, *p*<0.001; T2: *β*=0.657, *SE*=0.016, *p*<0.001). The CPP-predictor remained significantly predictive of action-update even when not controlling for main effect of PE*^h^* in the regression model (T1: *β*=0.881, *SE*=0.068, *p*<0.001; T2: *β*=0.958, *SE*=0.085, *p*<0.001). In contrast interaction effect with RU was only positively significant when CPP was included in the regression model (T1: *β_CPP-model_*=0.167; *SE*=0.036, *p*<0.001; *β_PE-model_*=-0.004; *SE*=0.023, *p*=0.877; T2: *β_CPP-model_*=0.182, *SE*=0.039, *p*<0.001; *β_PE-model=_*-0.014, *SE*=0.023, *p*=0.546). Moreover, the Hit regressor predicted the action-update negatively in both models (T1: *β_PE-model_*=-0.411, *SE*=0.016, *p*<0.001; *β_CPP-model_*=-1.163, *SE*=0.019, *p*<0.001; T2: *β_PE-model_*=-0.461, *SE*=0.021, *p*<0.001; *β_CPP-model_*=-1.128, *SE*=0.019, *p*<0.001). These separate regression models thus replicate the main findings from the combined model.

### Internal Consistency

We also examined the internal consistency of the beta weights gained from the separate regression models which was low for all normative predictors (T1: CPP: *r_SB_*=0.337, *95% CI* [0.238, 0.430]; RU*_PE-model_*: *r_SB_*=0.221, *95% CI* [0.116, 0.321]; RU*_CPP-model_*: *r_SB_*=0.306, *95% CI* [0.205, 0.400]; T2: CPP: *r_SB_*=0.438, *95% CI* [0.325, 0.540]; RU*_PE-model_*: *r_SB_*=0.403, *95% CI* [0.286, 0.509]; RU*_CPP-model_*: *r_SB_*=0.338, *95% CI* [0.215, 0.450]). The regression weights for PE*^h^* and Hit weights showed a good internal consistency (T1: PE*^h^*: *r_SB_*=0.942, *95% CI* [0.929, 0.953]; Hit*_PE-model_*: *r_SB_*=0.767, *95% CI* [0.718 0.808]; Hit*_CPP-model_: r_SB_*=0.875, *95% CI* [0.847, 0.898]; T2: PE*^h^*: *r_SB_*=0.963, *95% CI* [0.951, 0.971]; Hit*_PE-model_*: *r_SB_*=0.829, *95% CI* [0.783, 0.866]; Hit*_CPP-model_*: *r_SB_*=0.894, *95% CI* [0.864, 0.918]). Thus, overall this analysis replicated the main findings reported in the manuscript.

### Test-Retest Reliability

The test-retest reliability of the separate-model regression weights for the normative predictors were low (CPP: *ICC*=0.017, *95% CI* [-0.116, 0.148]; RU*_PE-model_*: *ICC*=0.000, *95% CI* [-0.132, 0.132]; RU*_CPP-model_*: *ICC*=0.119, *95% CI* [-0.014, 0.247]) while the weights for PE*^h^* and the Hit were moderate (PE*^h^*: *ICC*=0.727, *95% CI* [0.658, 0.784]; Hit*_PE-model_*: *ICC*=0.707, *95% CI* [0.633, 0.767]; Hit*_CPP-model_*: *ICC*=0.529, *95% CI* [0.427, 0.618]). This means, the psychometric quality of the beta weights from the separate models was not superior to the quality of the weights from the combined circular regression model reported in the main manuscript.

**Visual Inspections of Model-Predictions**

Finally, to assess the capabilities of the key variables CPP and Hit in predicting participants' action updates, we constructed two additional regression models alongside our full regression. The full model predicted action-update based on factors derived from the Bayesian learner (i.e., CPP and RU), their own accuracy (i.e., Hit), and PE. All predictors (except PE itself) were included as interaction effects with PE (cf. Supplemental Figure 8A). In contrast, the two additional models focused on individual variables. The CPP-model exclusively relied on CPP (cf. Supplemental Figure 8B), while the Hit-model solely utilized “Hit” as a regressor (cf. Supplemental Figure 8C).

We visually examined the relationship between the models' average predictions and the actual average action-updates across various PE sizes. To do so, we used the cut() function in R form the *base* package to bin the absolute PE (in radians) into 20 equally spaced bins. We then computed the average action-update and average model predictions within each bin across participants. The results, depicted in Supplemental Figure 8, highlight that the full model's predictions closely align with the overall shape of average action updates, demonstrating superior performance with a slight tendency to overestimate for larger prediction errors (PEs).

*
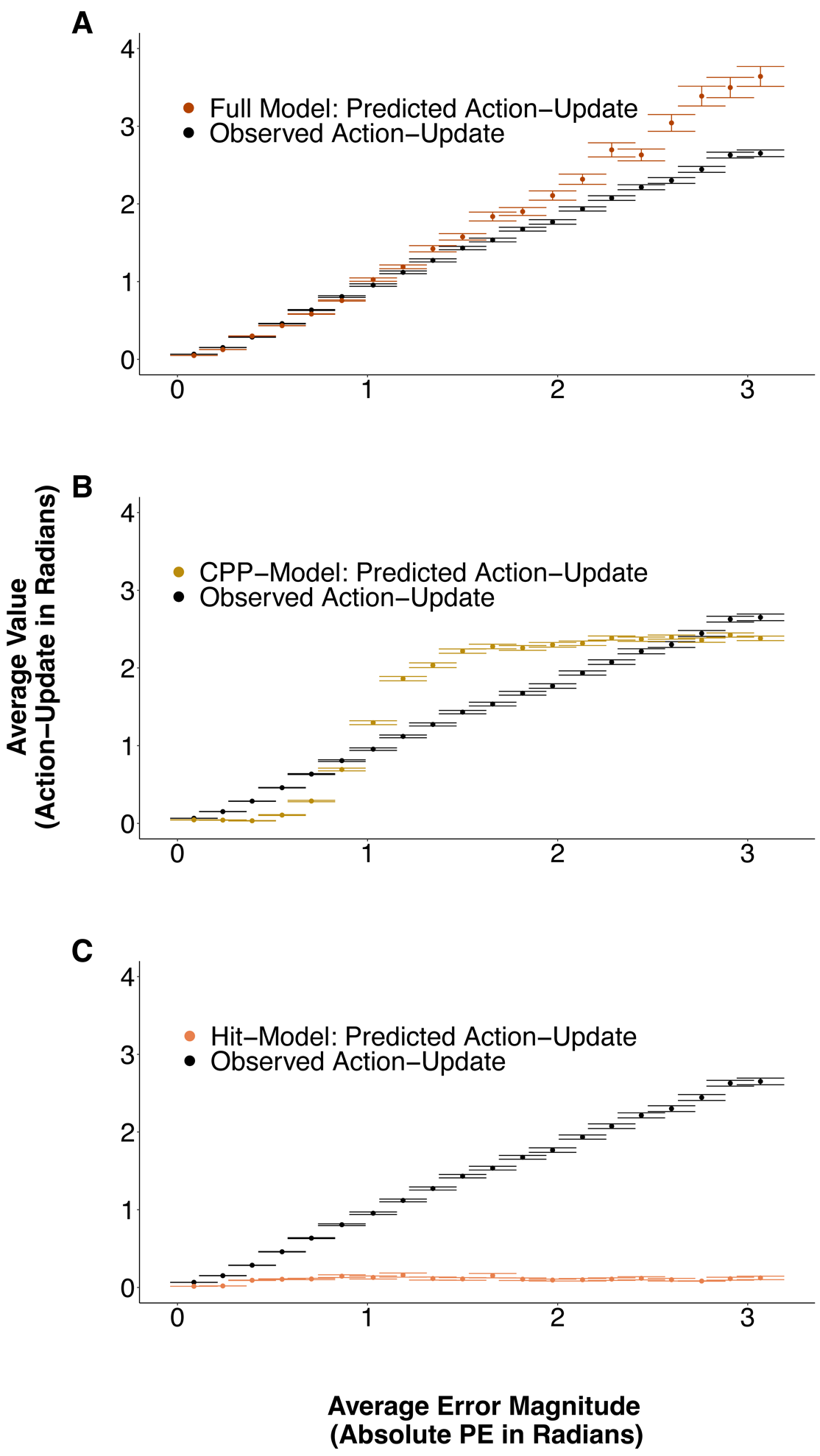
*

*Supplemental Figure 8:* Comparison of average regression model predictions with actual average action-updates across PE magnitudes. The full model's predictions closely mirror the overall shape of average action-updates, with a slight tendency to overestimate for large prediction errors (PEs; **A**). In contrast, CPP-model predictions display a sigmoid-like shape (**B**). The average predictions from the Hit-model form a shape reminiscent of a horizontal line, reflecting the fact that Hit remains at 0 beyond a certain PE size (**C**). Dots denote average action-updates and average model predictions across participants respectively for the corresponding error magnitude. Error bars indicate standard errors.

## Combined regression models predicting confidence ratings

For completeness, we also examined a combined linear regression model predicting confidence ratings with absolute PE*^h^*, CPP both included as predictors together with RU and Hit (cf. Main Manuscript).

PE*^h^*, CPP and RU were again negatively linked to confidence ratings (T1: *β_PE_^h^*=-0.108, *SE*=0.012, *p*<0.001; *β_CPP_*=-0.029, *SE*=0.012, *p*<0.05; *β_RU_*=-0.200, *SE*=0.007, *p*<0.001; T2: *β_PE_^h^*=-0.088, *SE*=0.016, *p*<0.001; *β_CPP_*=-0.067, *SE*=0.017, *p*<0.001; *β_RU_*=-0.197, *SE*=0.010, *p*<0.001) and Hit positively (T1: *β*=0.274, *SE*=0.008, *p*<0.001; T2: *β*=0.303, *SE*=0.011, *p*<0.001). This combined regression model thus replicates the confidence findings reported in the main manuscript.

### Internal Consistency

We also examined the internal consistency of the beta weights of this combined regression model which were again low for PE*^h^* (T1: *r_SB_*=0.021, *95% CI* [-0.087, 0.128]; T2: *r_SB_*=0.173, *95% CI* [0.041, 0.298]) and CPP (T1: *r_SB_*=0.207, *95% CI* [0.102, 0.308]; T2: *r_SB_*=0.314, *95% CI* [0.190, 0.429]) but good for RU (T1: *r_SB_*=0.802, *95% CI* [0.760,1 0.838]; T2: RU: *r_SB_*=0.850, *95% CI* [0.808, 0.883]) and Hit (T1: *r_SB_*=0.684, *95% CI* [0.622, 0.738]; T2: *r_SB_*=0.745, *95% CI* [0.679, 0.799]).

### Test-Retest Reliability

The test-retest reliability of the combined-model regression weights were again low (PE*^h^*: *ICC*=0.134, *95% CI* [0.003, 0.261]; CPP: *ICC*=0.231, *95% CI* [0.103, 0.351]; RU: *ICC*=0.467, *95% CI* [0.357, 0.564]; Hit: *ICC*=0.437, *95% CI* [0.324, 0.538]). This replicates the psychometric properties of the separate regression model beta weights reported in the main manuscript.

## Linear regression models predicting the non-circular action-update

We also investigated whether our findings would remain the same if we computed the regression models linking action-update and the Bayesian learner, in a similar way as Vaghi and colleagues (2017), i.e. by using the shortest total distance between the bucket positions to compute the action-update and the alternative approximation of the Bayesian learner (cf. Supplemental Methods). We also included the alternative regressor RU instead of RU (cf. Supplemental Methods).

The linear regression models again showed that action-update was positively linked to PE*^b^* (T1: *β_PE_^b^*=0.564, *SE*=0.017, *p*<0.001; T2: *β_PE_^b^*=0.627, *SE*=0.019, *p*<0.001) and that this link was positively modulated by CPP (*β*=0.347, *SE*=0.018, *p*<0.001; T2: *β*=0.318, *SE*=0.020, *p*<0.001) and RU (T1: *β*=0.039, *SE*=0.003, *p*<0.001; T2: *β*=0.026, *SE*=0.003, *p*<0.001). This alternative conceptualization of action-update was also negatively linked to Hit (T1: *β*=-0.028, *SE*=0.002, *p*<0.001; T2: *β*=-0.038, *SE*=0.003, *p*<0.001; cf. Supplemental Figure 9A). Thus, our main behavioural findings replicated when using the alternative way of approximating action-update and the normative factors.

### Internal Consistency

The internal consistency of these alternative regression weights of PE*^b^*, CPP and Hit was moderate (T1: PE*^b^: r_SB_*=0.653, *95% CI* [0.586, 0.710]; CPP: *r_SB_*=0.642, *95% CI* [0.574, 0.701]; Hit: *r_SB_*=0.589, *95% CI* [0.514, 0.656]; T2: PE*^b^*: *r_SB_*=0.758, *95% CI* [0.695, 0.809], CPP: *r_SB_*=0.731, *95% CI* [0.662, 0.787]; Hit: *r_SB_*=0.759, *95% CI* [0.696, 0.810]) while the internal consistency of the beta weights of RU was low (T1: *r_SB_*=0.266, *95% CI* [0.163, 0.364]; T2: *r_SB_*=0.265, *95% CI* [0.137, 0.384]; cf. Supplemental Figure 9B). Thus, overall the CPP regressor showed a better consistency in this alternative model but the consistency of PE*^b^* and Hit was lower than the consistency of their corresponding regressors in the main analysis.

### Test-Retest Reliability

Test-retest reliability was low to moderate for all regression weights (PE*^b^*: *ICC*=0.506, *95% CI* [0.399, 0. 599]; CPP: *ICC*=0.487, *95% CI* [0.380, 0.582]; RU: *ICC*=0.208, *95% CI* [0.080, 0.329]; Hit: *ICC*=0.467, *95% CI* [0.351, 0.568]; cf. Supplemental Figure 9C). Overall, the psychometric properties of the links captured by this alternative regression model were similar to the ones reported in the main manuscript.

*
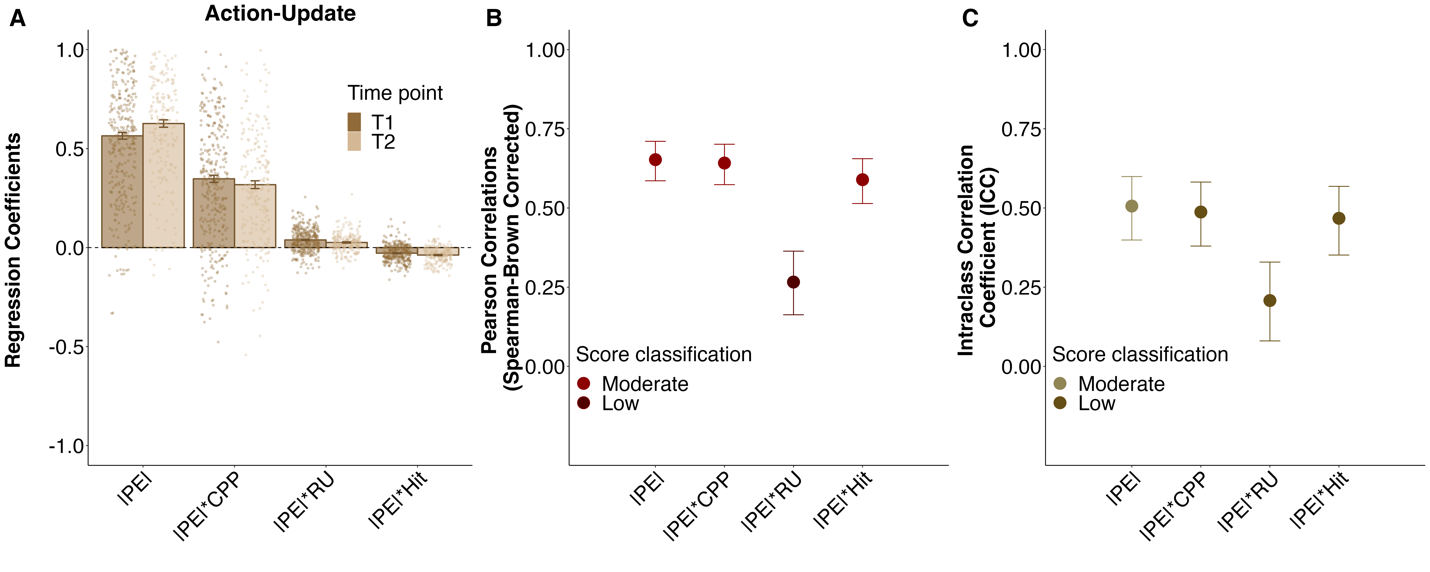
*

*Supplemental Figure 9.* Behavioural and psychometric properties of the link between action-update and the alternative Bayesian learner. Regression models for each participant predicted trial-wise action-updates (shortest linear distance) based on the model-derived normative factors PE*^b^* (model prediction error), CPP (change-point probability), RU (alternative implementation of RU) and Hit (accuracy on the previous trial). Action-update was positively linked to PE*^b^*, CPP, and RU and negatively linked to Hit **A**). All links showed a moderate internal consistency except for the RU interaction whose beta values had a low consistency (**B**). Test-retest reliability of all links were low, except for the one associated with PE*^b^*, which was moderate (**C**). In A, individual coefficients of participants are represented by circles, while the bar plot represents the mean of the model coefficients, and their error bars represent standard errors. Internal consistency for all measures is here displayed for T1. Error bars for internal consistency and test-retest reliability represent the estimates 95% confidence interval in.

## Confidence and the alternative Bayesian learner

Examining different analyses approaches used in the literature, we also ran regression models predicting confidence ratings on the basis of the alternative Bayesian factors and regressors. In contrast to previous studies, we again implemented separate regressions for PE*^b^* and CPP as done in the main manuscript.

Participants’ confidence ratings were negatively linked to all normative factors. PE*^b^* (T1: *β_PE_^b^*=-0.331, *SE*=0.011, *p*<0.001; T2: *β_PE_^b^*=-0.337, *SE*=0.013, *p*<0.001), CPP (*β*=-0.385, *SE*=0.014, *p*<0.001; T2: *β*=-0.388, *SE*=0.017, *p*<0.001) and RU (T1: *β_PE-model_*=-0.213, *SE*=0.008, *p*<0.001; *β_CPP-model_*=-0.275, *SE*=0.011, *p*<0.001; T2: *β_PE-model_*=-0.213, *SE*=0.011, *p*<0.001; *β_CPP-model_*=-0.273, *SE*=0.013, *p*<0.001). Accuracy on the preceding trial (i.e. Hit) predicted confidence positively (T1: *β_PE-model_*=0.169, *SE*=0.008, *p*<0.001; *β_CPP-model_*=0.154, *SE*=0.008, *p*<0.001; T2: *β_PE-model_*=0.193, *SE*=0.010, *p*<0.001; *β_CPP-model_*=0.180, *SE*=0.010, *p*<0.001; cf. Supplemental Figure 10A). Thus, the main findings reported in our main manuscript replicated when adapting this alternative version of the Bayesian learner.

### Internal consistency

We also examined the internal consistency of these alternative regression weights and saw that it was moderate to good across predictors (T1: PE*^b^: r_SB_*=0.730, *95% CI* [0.676, 0.777]; CPP: *r_SB_*=0.774, *95% CI* [0.727, 0.814]; RU*_PE-model_*: *r_SB_*=0.706, *95% CI* [0.647, 0.756]; RU*_CPP-model_*: *r_SB_*=0.767, *95% CI* [0.719, 0.808]; Hit*_PE-model_*: *r_SB_*=0.547, *95% CI* [0.467, 0.619]; Hit*_CPP-model_*: *r_SB_*=0.586, *95% CI* [0.511, 0.653]; T2: PE*^b^*: *r_SB_*=0.683, *95% CI* [0.605, 0.748], CPP: *r_SB_*=0.779, *95% CI* [0.721, 0.826]; RU*_PE-model_*: *r_SB_*=0.721, *95% CI* [0.650, 0.779]; RU*_CPP-model_*: *r_SB_*=0.829, *95% CI* [0.782, 0.866]; Hit*_PE-model_*: *r_SB_*=0.624, *95% CI* [0.536, 0.699]; Hit*_CPP-model_*: *r_SB_*=0.671, *95% CI* [0.591, 0.738]; cf. Supplemental Figure 10B). Thus, overall, the internal consistency of this alternative regression model was in line with our main model.

### Test-retest reliability

Test-retest reliability of the alternative regression weights were also similar to the reliability of the links reported in the main manuscript. The ICC sores showed that the weights of all regressors from the CPP-model had a low test-retest reliability (CPP: *ICC*=0.440, *95% CI* [0.327, 0.541]; RU: *ICC*=0.489, *95% CI* [0.382, 0.584]; Hit: *ICC*=0.374, *95% CI* [0.255, 0.483]), similarly to the weights of the PE-model (PE*^b^*: *ICC*=0.332, *95% CI* [0.209, 0.445]; RU: *ICC*=0.416, *95% CI* [0.300, 0.520]; Hit: *ICC*=0.318, *95% CI* [0.194, 0.432]; cf. Figure 6C).

*
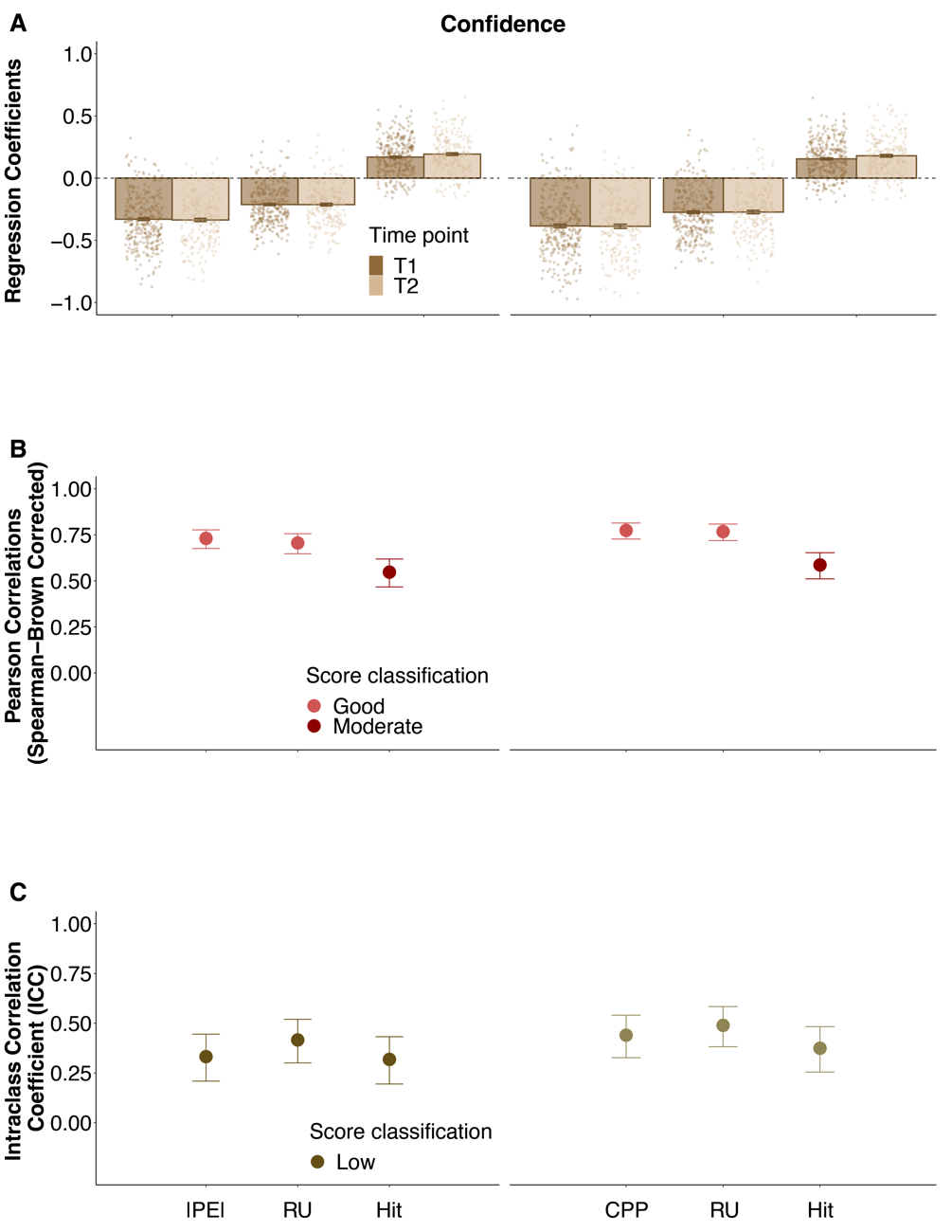
*

*Supplemental Figure 10.* Behavioural and psychometric properties of the link between confidence and the alternative Bayesian learner. Separate regression models were run for PE*^b^* (left-side of each plot) and CPP (right-side of each plot). Normative regressors negatively predicted confidence while accuracy (Hit) predicted it positively (**A**). Investigating the robustness of these associations, we saw that the internal consistency of the links between confidence and normative regression weights was moderate to good (Spearman-Brown corrected Pearson correlations; **B**). Test-retest reliability of all normative factor regression weights predicting confidence was low (as indicated by the ICC score; **C**). In A, individual coefficients of participants are represented by circles, while the bar plot represents the mean of the model coefficients, and their error bars represent standard errors. Internal consistency for all measures is here displayed for T1. Error bars for internal consistency and test-retest reliability represent the estimates 95% confidence interval.

### Associations between task measures and psychiatric dimension scores

In line with the findings reported in the main manuscript, OC scores were not significantly correlated with PE*^b^* regression weights gained from this alternative action-update model (*r_S_*=0.093, *p=*0.169). Similarly, none of the psychiatric symptoms showed an association with the mean unsigned, non-circular version of LR*^h^* (OC symptoms: *r_S_*=-0.013, *p=*0.852; anxiety: *r_S_*=0.037, *p=*0.582; depression: *r_S_*=-0.039, *p=*0.566).

## Mixed-models examining the link between behaviour and the Bayesian learner

### Action-update and the adapted Bayesian learner

We ran a mixed-effects model predicting action-update (approximated as the shortest linear distance) on the basis of the Bayesian learner’s predictions to replicate analyses conducted by Seow & Gillan (2020). This model again showed that action-update was positively linked to all normative factors at both time points (T1: *β_PE_^b^*=0.563, *SE*=0.017, *p*<0.001; *β_CPP_*=0.350, *SE*=0.018, *p*<0.001; *β*_RU_=0.039, *SE*=0.003, *p*<0.001; T2: *β_PE_^b^*=0.625, *SE*=0.019, *p*<0.001; *β_CPP_*=0.320, *SE*=0.020, *p*<0.001; *β*_RU_=0.026, *SE*=0.003, *p*<0.001) and negatively linked to Hit (T1: *β*=-0.029, *SE*=0.002, *p*<0.001; T2: *β*=-0.038, *SE*=0.003, *p*<0.001; cf. Supplemental Figure 11A).

#### Internal Consistency

We also examined the internal consistency of these mixed-model links between action-update the normative factors and the Hit predictor, which were moderate to good (T1: PE*^b^*: *r_SB_*=0.782, *95% CI* [0.736, 0.821]; CPP: *r_SB_*=0.660, *95% CI* [0.594, 0.717]; RU: *r_SB_*=0.537, *95% CI* [0.455, 0.609]; Hit: *r_SB_*=0.664, *95% CI* [0.599, 0.720]; T2: PE*^b^*: *r_SB_*=0.841, *95% CI* [0.797, 0.876]; CPP: *r_SB_*=0.802, *95% CI* [0.749, 0.845]; RU: *r_SB_*=0.676, *95% CI* [0.597, 0.742]; Hit: *r_SB_*=0.596, *95% CI* [0.503, 0.675]; cf. Supplemental Figure 11B).

#### Test-Retest Reliability

Test-retest reliability of the mixed-model regression weights capturing the link between normative factors and action-update were low to moderate (PE*^b^*: *ICC*=0.572, *95% CI* [0.449, 0.669]; CPP: *ICC*=0.563, *95% CI* [0.464, 0.647]; RU: *ICC*=0.339; *95% CI* [0.034, 0.553]; Hit: *ICC*=0.379, *95% CI* [0.209, 0.517]; cf. Supplemental Figure 11C).

### Confidence and the adapted Bayesian learner

We repeated the same mixed-effects models as above now predicting confidence ratings. We again observed that participants’ confidence was negatively linked to all of the normative factors (T1: *β_PE_^b^*=-0.150, *SE*=0.010, *p*<0.001; *β_CPP_*=-0.267, *SE*=0.014, *p*<0.001; *β*_RU_=-0.283, *SE*=0.010, *p*<0.001; T2: *β_PE_^b^*=-0.150, *SE*=0.012, *p*<0.001; *β_CPP_*=-0.269, *SE*=0.018, *p*<0.001; *β*_RU_=-0.281, *SE*=0.013, *p*<0.001), and positively to Hit (T1: *β*=0.129, *SE*=0.008, *p*<0.001; T2: *β*=0.155, *SE*=0.011, *p*<0.001; cf. Supplemental Figure 11D).

#### Internal consistency

We also examined the internal consistency of the links revealed by the mixed-model and again saw that across predictors it was moderate to good (T1: PE*^b^: r_SB_*=0.773, *95% CI* [0.726, 0.813]; CPP: *r_SB_*=0.429, *95% CI* [0.814, 0.876]; RU: *r_SB_*=0.549, *95% CI* [0.832, 0.888]; Hit: *r_SB_*=0.630, *95% CI* [0.560, 0.691], T2: CPP: *r_SB_*=0.787, *95% CI* [0.730, 0.832]; RU: *r_SB_*=0.892, *95% CI* [0.862, 0.916]; Hit: *r_SB_*=0.714, *95% CI* [0.642, 0.773]) except for PE*^b^* at the second time point which showed a low internal consistency (PE*^b^*: *r_SB_*=0.344, *95% CI* [0.222, 0.456]; cf. Supplemental Figure 11E).

#### Test-retest reliability

Test-retest reliability of the mixed-effects regression weights of the normative factors predicting confidence was low to moderate (PE*^b^*: *ICC*=0.000, *95% CI* [-0.129, 0.130]; CPP: *ICC*=0.471, *95% CI* [0.362, 0.568]; RU: *ICC*=0.508, *95% CI* [0.403, 0.600]; Hit: *ICC*=0.389, *95% CI* [0.271, 0. 495]; cf. Supplemental Figure 11F).


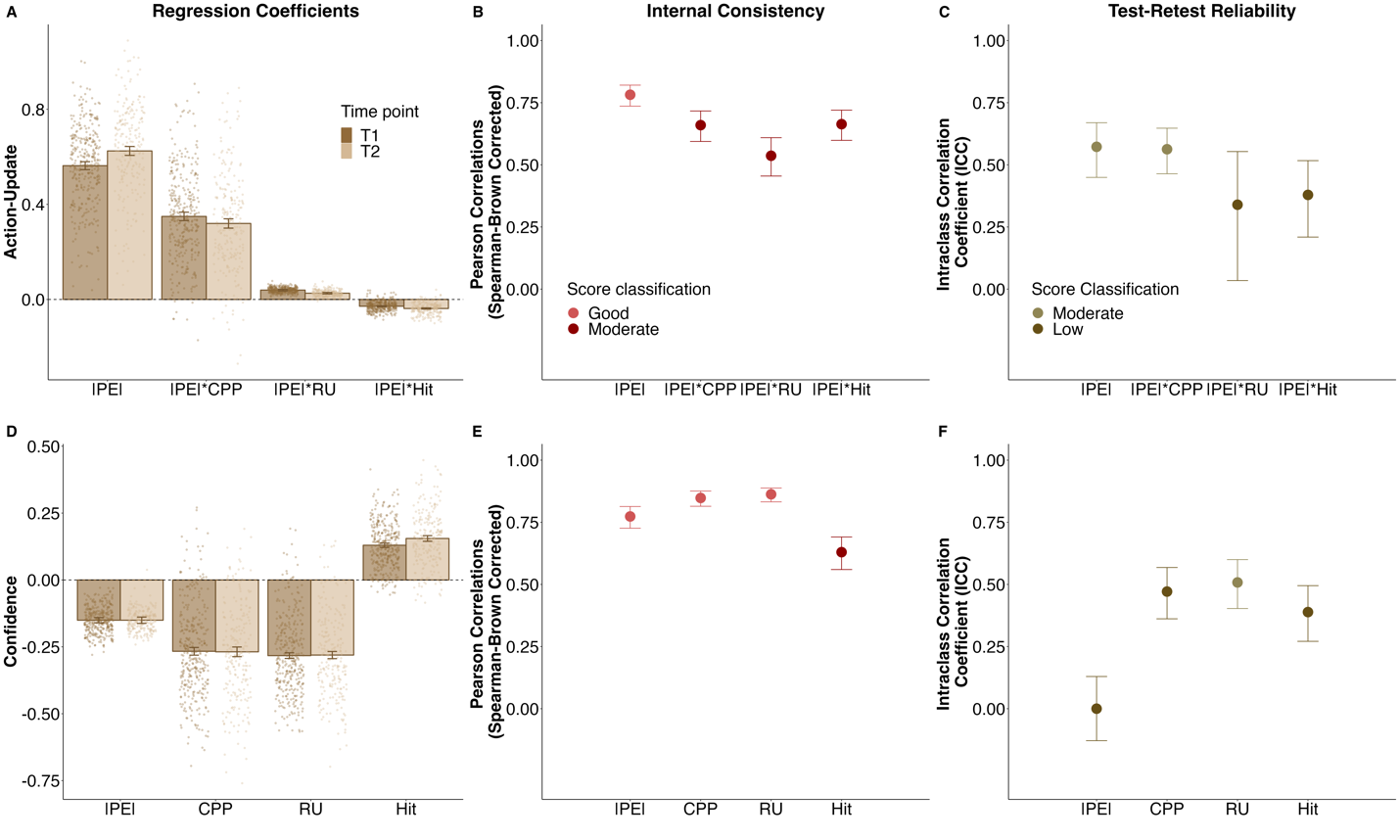


Supplemental Figure 11. Mixed-effect models results predicting confidence and action on the basis of the alternative Bayesian factors. Regression models predicted trial-wise action-updates (shortest absolute distance) and confidence ratings based on the model-derived normative factors PE^b^ (model prediction error), CPP (change-point probability), RU (relative uncertainty) and Hit (accuracy on the previous trial). All normative factors were positively and the Hit regressor negatively linked to action-update (**A**). Investigating the robustness of these associations, we saw that the internal consistency of the regression weights were moderate to good (**B**). Test-retest reliability of all regression weights predicting action-update were low to moderate (**C**). We found the reverse relationships for confidence, which was negatively linked to the normative factors and positively linked to Hit (**D**). Internal consistency of the regression weights was again moderate to good (**E**). All regression weights in the action-update model showed a low to moderate test-retest reliability (**F**). Individual coefficients of participants are represented by circles while the bar plot represents the overall model coefficient, and their error bars represent standard errors in A & D. Internal consistency for all measures is here displayed for T1. Error bars for internal consistency and test-retest reliability represent the estimates 95% confidence interval.

### Mixed-models investigating associations between task measures and psychiatric dimension scores

For replication purposes, we also examined mixed-effects models that had been previously implemented by Seow and Gillan (2020) and which investigated the link between confidence, action-update and psychiatric dimension scores (i.e. OC symptoms, anxiety, and depression; cf. Supplemental Methods above).

We again did not observe any link between any psychiatric symptom score and action-confidence coupling. The interaction effects between the psychiatric scores and confidence in the regression models predicting action-update were non-significant for all symptom scores (OC symptoms: *β=*-0.076, *SE*=0.376, *p*=0.840; anxiety: *β*=-0.180, *SE*=0.376, *p*=0.632; depression: *β*=-0.197, *SE*=0.376, *p*=0.600). Thus, we still could not replicate the association between action-confidence coupling and OC or any other psychiatric symptom score when repeating the mixed-effects models.

Similarly, none of the psychiatric variables in these models were predictive of confidence (OC symptoms: *β*=-0.000, *SE*=0.005, *p*=1; anxiety: *β*=0.000, *SE*=0.005, *p*=1; depression: *β*=0.000, *SE*=0.005, *p*=1) or action-update themselves (OC symptoms: *β*=0.262, *SE*=0.437, *p*=0.550; anxiety: *β*=0.035, *SE*=0.306, *p*=0.908; depression: *β*=-0.319, *SE*=0.275, *p*=0.247).

Moreover, none of the psychiatric variables showed a significant interaction effect with CPP on confidence (OC symptoms: *β*=0.013, *SE*=0.016, *p*=0.422; anxiety: *β*=-0.006, *SE*=0.016, *p*=0.667; depression: *β*=-0.004, *SE*=0.016, *p*=0.787) and we also did not find that OC scores affected the impact of PE*^b^* on action-update (*β*=-0.004, *SE*=0.019, *p*=0.818) using the mixed-model approach.

# Stability of additional associations investigated in previous research

## Association between action-update and PE^h^

As indicated by the analyses in the main manuscript, participants' action-update was linked to their own errors (i.e. PE*^h^*) which was underlined by high circular correlations between the two measures at both time points before, at and after CPs (all $r_{circ}\geq$0.667; cf. Supplemental Figure 12A).

### Internal consistency

Although the link between participants' PE*^h^* and action-update was clear at both time points, the correlations’ internal consistency was low throughout the investigated trials at both time points (cf. Supplemental Figure 12B). This is in contrast to the high internal consistency observed for the PE*^h^*-regression weights predicting action-update while statistically controlling for other task variables (all *r_SB_*$\leq0.489$; cf. Main Manuscript).

### Test-retest reliability

Similarly, test-retest reliability of the circular regression coefficients capturing the link between PE*^h^* and action-update was low throughout all trials (*ICC*$\leq$0.233; cf. Supplemental Figure 12C).

## Association between action-update and RU

The link between action-update and RU, previously investigated in the literature (e.g. Nassar et al. 2019), was not only low in our data when captured by regression models controlling for additional task variables (cf. Main Manuscript) but also when measured by circular correlations before, at and after CPs (all $r_{circ}\leq$0.039; cf. Supplemental Figure 12D).

### Internal consistency

The internal consistency was low throughout the investigated trials (all *r_SB_*$\leq0.108$; cf. Supplemental Figure 12E).

### Test-retest reliability

Test-retest reliability of the circular correlation between action-update and RU and the investigated trials was also low (all *ICC*$\leq$0.078; cf. Supplemental Figure 12F).


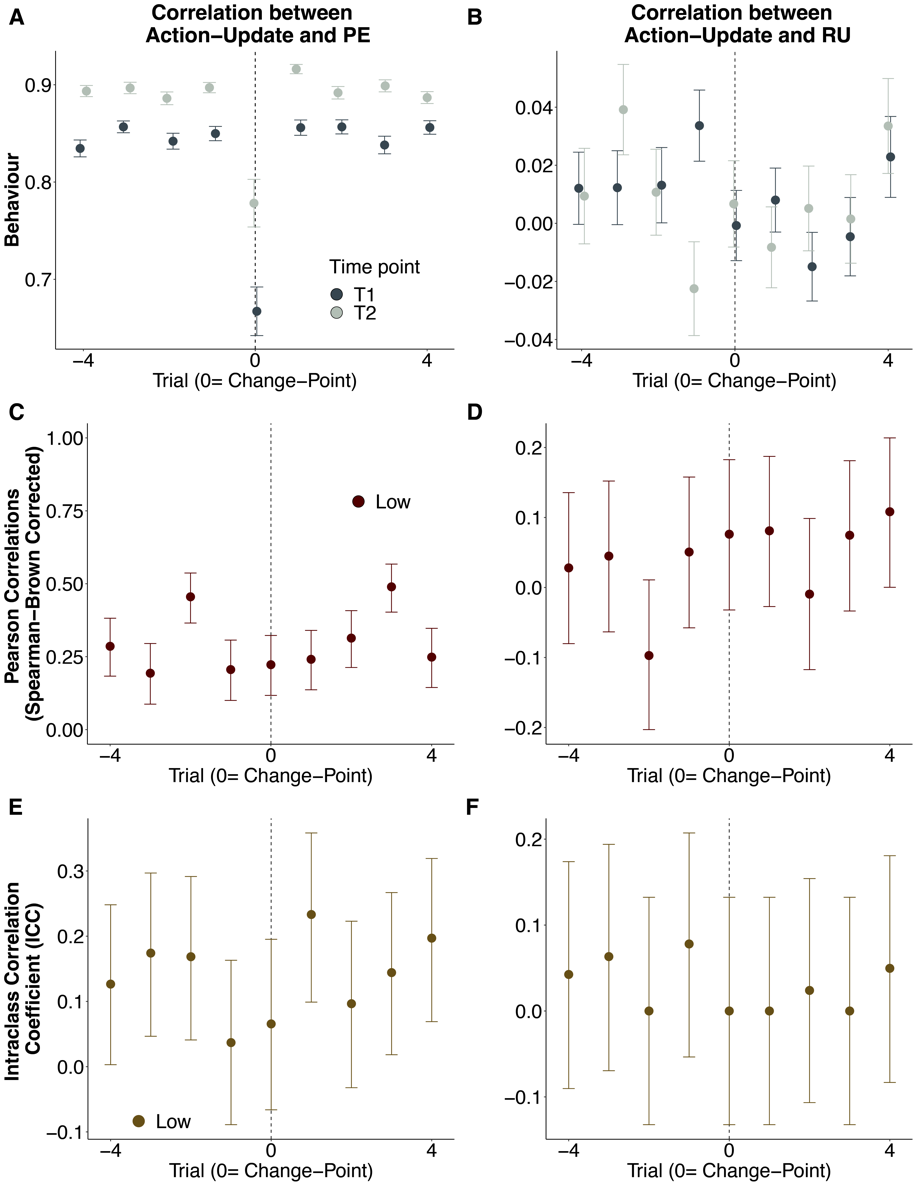


Supplemental Figure 12. The link between action-update and PE^h^ and RU and the associated psychometric properties. Participants’ action-update at time point 1 (T1; N_T1_=330) and 2 (T2; N_T2_=219) was highly correlated with their own errors (PE^h^) before at and after CPs (**A**). Action-update was not correlated with uncertainty in the task captured by the normative factor RU (**D**). Internal consistency (Spearman-Brown corrected Pearson correlations) for correlation between action-update and PE^h^ (**B**) and action-update and RU (**E**) at all investigated trials before, at, and after CPs was, however, low (here displayed for T1; **B**). Test-retest reliabilities measured by ICC-scores were also low for all investigated correlations (**C & F**). Error bars represent standard errors in A & D. Error bars for the remaining plots represent the estimates 95% confidence interval.

## Association between the LR*^h^* and the distance to the preceding CP

Finally, we also investigated the model free version of the relative uncertainty term of the Bayesian learner by computing the correlation between the LR*^h^* and the inverse of the distance to the preceding CP ($i.e. \frac{1}{number of trials since the last CP}$). This association was only significant at the second time point (T1: *r_S_*=0.069, *p=*0.081; T2: *r_S_*=0.190, *p<*0.001) suggesting participants adapted their error-driven behaviour more relative to the distance to a change in the task when they played it for the second time.

### Internal consistency

The internal consistency of this link was moderate at the first time point (*r_SB_*=0.526, *95% CI* [0.443, 0.600]) and good at the second time point (*r_SB_*=0.758, *95% CI* [0.683, 0.801]).

### Test-retest reliability

Test-retest reliability of the link between LR*^h^* and the inverse distance to the last CP was moderate (*ICC*=0.326, *95% CI* [0.204, 0.439]).

# Covid-adapted OC symptom score and the predictive-inference task.

Since the present study was conducted during the Covid-19 pandemic, we created a new subscore for the Obsessive Compulsive Inventory-Revised (OCI-R; Foa et al. 2002) (i.e. ‘OCI-R pandemic-irrelevant items’) excluding items of the OCI-R that we considered being potentially related to or influenced by the Covid-19 pandemic (cf. Supplemental Table 2).

We subsequently repeated all main analyses, investigating the link between OC symptoms and task, behavioural, and normative parameters now using the OCI-R pandemic-irrelevant items score instead of the regular OCI-R total score as a predictor.

First, we investigated the relationship between action-confidence coupling and the new OCI-R score. However, the action-confidence beta weights did not correlate with the OC pandemic-irrelevant score (*r_S_*=0.005, *p=*0.941). Similarly, the OCI-R pandemic-irrelevant items score was not correlated with mean confidence (*r_S_*=-0.013, *p=*0.851) or absolute action-update (*r_S_*=0.012, *p=*0.855).

We also repeated the analyses linking the CPP-weights predicting confidence and the new OCI-R score, but it was nonsignificant (*r_S_*=0.027, *p=*0.693). Similarly, in the action-update model the new OCI-R score was not associated with the PE*^b^*-weights (*r_S_*=0.087, *p=*0.201).

Finally, the new OCI-R score also was not correlated with the mean absolute LR*^h^* (*r_S_*=0.006, *p=*0.931). Overall, this means, that even when controlling for potential inflations of the OCI-R scores due to the pandemic situation we could not replicate any of the associations between OC symptoms and task measures reported in the literature (these findings held true when adapting the mixed-model approach reported above).

**Supplemental Table 2**

*Pandemic-relevancy of items on the OCI-R*

| Pandemic-relevance | Item |
| --- | --- |
| No | 1. I have saved up so many things that they get in the way. |
| No | 1. I check things more often than necessary. |
| No | 1. I get upset if objects are not arranged properly. |
| No | 1. I feel compelled to count while I am doing things. |
| Yes | 1. I find it difficult to touch an object when I know it has been touched by strangers or certain people. |
| No | 1. I find it difficult to control my own thoughts. |
| No | 1. I collect things I don’t need. |
| No | 1. I repeatedly check doors, windows, drawers, etc.. |
| No | 1. I get upset if others change the way I have arranged things. |
| No | 1. I feel I have to repeat certain numbers. |
| Yes | 1. I sometimes have to wash or clean myself simply because I feel contaminated. |
| No | 1. I am upset by unpleasant thoughts that come into my mind against my will. |
| No | 1. I avoid throwing things away because I am afraid I might need them later. |
| No | 1. I repeatedly check gas and water taps and light switches after turning them off. |
| No | 1. I need things to be arranged in a particular order. |
| No | 1. I feel that there are good and bad numbers. |
| Yes | 1. I wash my hands more often and longer than necessary. |
| No | 1. I frequently get nasty thoughts and have difficulty in getting rid of them. |

# Comparison of psychiatric symptom and behavioural measures’ distributions

To further investigate why we did not replicate previous findings reported by Seow and Gillan (2020), we carefully compared symptom and behavioural measures’ distributions in our and Seow and Gillan’s (2020) sample in post-hoc exploratory analyses.

Looking at OCI-R total scores, using a simple two-sided t-test, we observed a trend for a difference between the two samples on the OCI-R total score (*t*(463.92)=1.844, *p*=0.066; cf. Supplemental Figure 13A), which vanished when using the adapted OCI-R score that controlled for items that might have been of particular relevance to the ongoing pandemic (*t*(456.38)=1.097, *p*=0.273; cf. Supplemental Figure 13B).

We also compared the mean behavioural scores of the two main variables of interest, confidence and LR*^h,^* across the two studies. As Seow and Gillan’s study entailed two different hazard rates (H=0.025 and H=0.125), for the analyses below we only used trials in their data with the same hazard rate as ours (H=0.125). While for our T1 sample only the mean LR*^h^* was higher than in the sample of Seow and Gillan (Confidence: *t*(755.67)=-0.721, *p*=0.471; LR*^h^*: *t*(659.95)=7.668, *p*<0.001), both, mean confidence and mean LR*^h^* were higher for our T2 sample (Confidence: *t*(518.16)=2.563, *p*=0.011; LR*^h^*: *t*(462.81)=3.699, *p*<0.001; cf. Supplemental Figure 13C & D).

Approximately 50% of participants in Seow and Gillan’s study had completed the trials matching our hazard rate at the beginning of the task, while the other half had first played trials with the different hazard rate. Controlling for potential biasing effects caused by the encounter of different hazard rates, we thus conducted a follow-up analysis only including Seow and Gillan’s participants that had played our hazard rate at the beginning of the task. In this analysis, the difference in mean confidence and mean LR*^h^* between their and our T2 sample turned non-significant (Confidence: *t*(418.66)=1.471, *p*=0.142); LR*^h^*: *t*(412.1)=1.685, *p*=0.093). This was even though the Seow and Gillan’s main findings remained true when using this adapted dataset in their analyses.

Overall, the mentioned analyses do not explain the difference in findings we observed as OCI-R scores and task measures are not strikingly different in our samples. This difference in findings across studies further stays in contrast with our main findings showing the robustness of the task measures across time and within task sessions in our sample (cf. Discussion section of the main manuscript for potential explanations for this difference in findings).

**

*Supplemental Figure 13.* Distributions of OCI-R scores, LR^h^ and confidence in the present study and the sample reported by Seow and Gillan (2020). While regular total scores of the regular Obsessive-Compulsive Inventory-Revised (OCI-R) showed a trend-level difference between samples (**A**), the newly created OCI-R subscore only including pandemic-irrelevant items did not differ across samples (**B**). Our sample (N=219) showed overall higher mean confidence (**C**) and learning rate (**D**) in the predictive-inference task than the sample reported by Seow and Gillan (2020) (N=437). Task measures displayed are from time point 2, while learning rate patterns were the same at both time points mean confidence between the samples was not significantly different at time point 1. Dots represent total scores of individual participants. Paired t-test (two-tailed): t.=trend, n.s.=non-significant. Plots have been created using the raincloud package in R *(Allen et al., 2021)*.

Supplemental References

Allen, M., Poggiali, D., Whitaker, K., Marshall, T. R., van Langen, J., & Kievit, R. A. (2021). Raincloud plots: A multi-platform tool for robust data visualization. *Wellcome Open Research*, *4*, 63. https://doi.org/10.12688/wellcomeopenres.15191.2

Bates, D., Kliegl, R., Vasishth, S., & Baayen, H. (2018). Parsimonious Mixed Models. *arXiv:1506.04967 [Stat]*. http://arxiv.org/abs/1506.04967

Bates, D., Mächler, M., Bolker, B., & Walker, S. (2015). Fitting Linear Mixed-Effects Models Using lme4. *Journal of Statistical Software*, *67*(1), 1–48. https://doi.org/10.18637/jss.v067.i01

Foa, E. B., Huppert, J. D., Leiberg, S., Langner, R., Kichic, R., Hajcak, G., & Salkovskis, P. M. (2002). The Obsessive-Compulsive Inventory: Development and validation of a short version. *Psychological Assessment*, *14*(4), 485–496.

McGuire, J. T., Nassar, M. R., Gold, J. I., & Kable, J. W. (2014). Functionally Dissociable Influences on Learning Rate in a Dynamic Environment. *Neuron*, *84*(4), 870–881. https://doi.org/10.1016/j.neuron.2014.10.013

Nassar, M. R., Bruckner, R., & Frank, M. J. (2019). Statistical context dictates the relationship between feedback-related EEG signals and learning. *eLife*, *8*, e46975. https://doi.org/10.7554/eLife.46975

Nassar, M. R., Bruckner, R., Gold, J. I., Li, S.-C., Heekeren, H. R., & Eppinger, B. (2016). Age differences in learning emerge from an insufficient representation of uncertainty in older adults. *Nature Communications*, *7*, 11609. https://doi.org/10.1038/ncomms11609

Nassar, M. R., Waltz, J. A., Albrecht, M. A., Gold, J. M., & Frank, M. J. (2021). All or nothing belief updating in patients with schizophrenia reduces precision and flexibility of beliefs. *Brain*, *144*(3), 1013–1029. https://doi.org/10.1093/brain/awaa453

Nassar, M. R., Wilson, R. C., Heasly, B., & Gold, J. I. (2010). An Approximately Bayesian Delta-Rule Model Explains the Dynamics of Belief Updating in a Changing Environment. *Journal of Neuroscience*, *30*(37), 12366–12378. https://doi.org/10.1523/JNEUROSCI.0822-10.2010

Seow, T. X. F., & Gillan, C. M. (2020). Transdiagnostic Phenotyping Reveals a Host of Metacognitive Deficits Implicated in Compulsivity. *Scientific Reports*, *10*(1), Article 1. https://doi.org/10.1038/s41598-020-59646-4

Vaghi, M., Luyckx, F., Sule, A., Fineberg, N. A., Robbins, T. W., & De Martino, B. (2017). Compulsivity Reveals a Novel Dissociation between Action and Confidence. *Neuron*, *96*(2), 348-354.e4. https://doi.org/10.1016/j.neuron.2017.09.006
